# Development of a measure for patients preparing to start dialysis and their partners: The Starting Dialysis Questionnaire (SDQ)

**DOI:** 10.1186/s12955-020-01610-x

**Published:** 2020-11-07

**Authors:** Currie Moore, Alison Wearden, Lesley-Anne Carter, Sandip Mitra, Suzanne M. Skevington

**Affiliations:** 1 School of Health Sciences, Division of Psychological Sciences and Mental Health, Manchester Centre for Health Psychology, Coupland Building I, University of Manchester, Oxford Road, Manchester, M13 9PL UK; 2 Manchester Academic Health Science Centre, University of Manchester, Manchester, UK; 3 Division of Population Health, Health Services Research and Primary Care, University of Manchester, Manchester, UK; 4 Manchester University NHS Foundation Trust, Manchester, UK; 5 NIHR Devices for Dignity, MedTech Cooperative, Sheffield, UK

**Keywords:** End stage renal disease, Cognitive interview, Measure, Development, Expectations, Acceptance, Relationship, Couples, Dialysis

## Abstract

**Background:**

The transition onto dialysis is a stressful time that affects both patients and their partners. Research suggests that psychological and interpersonal characteristics within the couple are related to how well they adapt to dialysis. The aim of this multi-phase, mixed methods study was to develop a measure, the Starting Dialysis Questionnaire (SDQ), that is applicable to both patients and their partners and assesses their own thoughts and feelings about these constructs.

**Methods:**

Data from semi-structured interviews with patients and their partners (n = 22 couples) were analysed using theoretical thematic analysis to identify and define constructs related to quality of life (QOL). Next, items addressing these constructs were derived from the interviews. Then, cognitive interviews were conducted with patients with chronic kidney disease and their partners (n = 5 couples) to assess the face validity and comprehensibility of the items. Lastly, preliminary psychometric properties were evaluated in a sample of patients preparing to start dialysis and their partners (n = 83 couples).

**Results:**

Three themes related to QOL were identified, namely dialysis expectations, accepting dialysis and dyadic relationship characteristics. The cognitive interviews refined the SDQ and established its face validity. Psychometric assessments indicated that overall the items performed well and did not show significant floor or ceiling effects. Good internal consistency was found within the three domains, and items correlated within the domains.

**Conclusions:**

The SDQ is a measure (34 items) that assesses key psychological and interpersonal factors in patients and their partners as they start dialysis. It shows good preliminary psychometric properties; however, a large-scale field trial is needed to establish its validity. Once validated, it could offer a clinically useful tool to assist clinicians in preparing patients and partners for dialysis.

## Background

Dialysis is a treatment for people who are in end stage renal disease (ESRD) and whose kidneys can no longer eliminate toxins from the body. Starting dialysis has been identified as a stressful time in the treatment pathway for patients and also their family members, or partners [1]. Patients and their partners form a unique social unit, or dyad. Members of the dyad are mutually affected by treatments, and dyadic characteristics may also affect treatment and health-related outcomes [2].

Previous qualitative research found that psychological and interpersonal factors within the dyad played an important role in adjusting to dialysis and minimising the impact of it on the dyadic relationship and quality of life (QOL) [3]. Moore et al*.* [3] interviewed both members of 20 dyads at various times around the start of dialysis. Patients and their partners described significant changes in their identities, roles within the relationship and responsibilities. However, dyads who acted as a team buffered their relationship from these stressors by being positive, accepting dialysis or normalizing dialysis. Assessing these psychological and interpersonal characteristics in both patients and their partners as they prepare to start dialysis may identify dyads who would benefit from additional support over this transition.

A scoping review of the literature yielded 16 existing questionnaires which measured constructs related to relevant (on the basis of our previous qualitative work) [3] psychological and interpersonal factors (7 on dyadic coping, adjustment or satisfaction, 6 on illness cognition, 1 on couples’ communication, 1 on optimism and 1 on expectations for QOL and health after renal transplants). Each questionnaire was reviewed on the basis of the following criteria: (1) it addressed psychological or interpersonal relationship dynamics, (2) it was amenable to changes in wording (e.g., if a general questionnaire about illness, it needed to be possible to change “illness” to “kidney disease” or “treatment” to “dialysis”) and (3) it was applicable to patients and their partners. The only questionnaire partly meeting these criteria was the couples’ communication questionnaire [4]; however, it only addresses one specific area related to interpersonal factors (i.e., communication). Therefore, this search indicated that no measure existed which assesses the psychological and interpersonal factors related to maintaining the dyadic relationship and QOL, as described by Moore et al*.* [3].

**Fig. 1:**
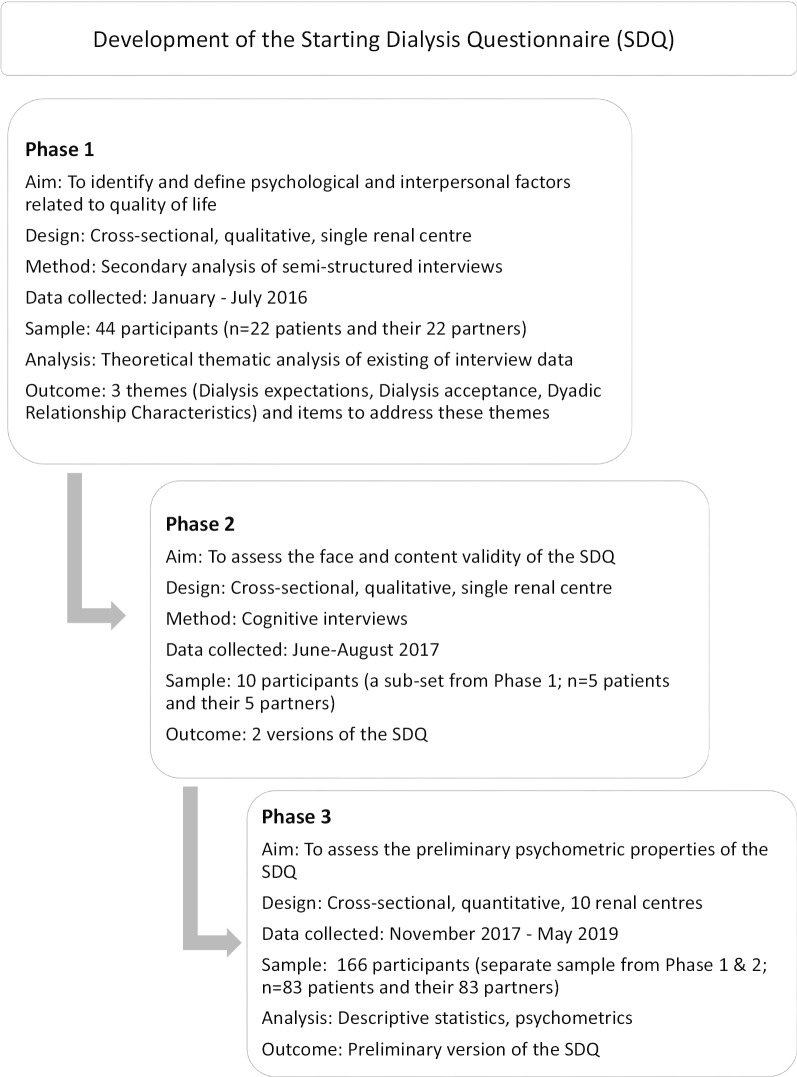
Characteristics of the phases of the questionnaire’s development

The present study aimed to fill this gap through the development of the Starting Dialysis Questionnaire (SDQ). The study presented here describes the three-phase development of the SDQ. Firstly, using existing interview data, the psychological and interpersonal factors relating to QOL were identified and defined through qualitative analysis, and then questions, or items, were generated from the interview data (Phase 1). Secondly, the items were assessed for comprehensibility and relevance in cognitive interviews (Phase 2). Thirdly, preliminary psychometric properties of the items and proposed domains of the SDQ were evaluated (Phase 3). Figure 1 provides an overview and key characteristics of each phase.

## Aims

***Phase 1: Identification of psychological and interpersonal factors related to QOL and generation of items***

- To identify and define key psychological and interpersonal factors that relate to QOL in patients and their partners during the early phases of dialysis.
- To develop items related to these factors using an inductive, data-driven approach.

***Phase 2: Refinement of the SDQ using cognitive interviews***

- To use cognitive interviewing to assess the comprehensibility and validity of the SDQ in a sample of patients with ESRD and their partners.

***Phase 3: Preliminary assessment of the psychometric properties of the SDQ***

- To assess basic psychometric properties of the SDQ in a sample of patients preparing to start dialysis and their partners.

## Methods

### Phase 1

#### Design

The data used in this phase of the study were part of an over-arching, cross-sectional, qualitative study which explored the impact of the early phases of dialysis on patients and their partners [3]. The early phases of dialysis were defined as the period when patients are preparing to start dialysis (e.g., pre-dialysis) and are adjusting to dialysis [1]. In this secondary analysis, we conducted a secondary analysis of all the semi-structured interview data.

#### Participants

Participants were recruited from a single renal unit in England. The inclusion criteria were that patients and partners were over 17 years old, spoke English fluently and were in a spousal-type relationship. Pre-dialysis patients were drawn from the hospital’s renal registers and had an estimated glomerular filtration rate (eGFR) of ≤ 20 (a clinical marker denoting how well the kidneys are removing toxins in the blood) but did not have a planned start date for dialysis. Patients adjusting to dialysis were defined as those utilising any form of outpatient haemodialysis (HD) or peritoneal dialysis (PD) for less than 16 months. This time frame (up to 16 months) is under-researched in the ESRD literature; however, our previous research found that psychological and interpersonal experiences were reported similarly across the phases of early dialysis and types of dialysis [3]. Purposive sampling was utilised to ensure that a range of experiences could inform the research question. The sampling framework focused on two patient characteristics, namely dialysis phase and type of dialysis.

#### Procedure

Face-to-face semi-structured interviews were conducted by the lead author (CM), lasting an average of 50 min (range 11–102 min). Four couples requested to be interviewed together, and two patients were present during their partners’ interview. The interviews followed a topic guide developed by the research team which included questions on QOL, factors related to QOL, their partners’ QOL and the impact on their relationship. Participants on dialysis were asked about their current experiences in relation to these topics and also how these were affected by starting dialysis. The interviews were audio-recorded and transcribed verbatim.

#### Qualitative analysis

In this analysis of the interviews, a theoretical thematic analysis [5, 6] was conducted to consolidate and define psychological and relationship factors related to QOL. The primary analysis of the data utilised an inductive, dyadic thematic analysis approach [5–7], a full account of the analysis and findings are reported in the corresponding publication [3]. Briefly, the analysis was conducted with a focus on the dyad, starting with the patient and followed by the partner in each dyad. The first step was familiarisation with the data (repeated readings of the interview transcripts) followed by line by line coding using inductively driven codes. The codes were then assimilated into working themes and transferred onto a chart which captured the experiences of the dyads across key topics related to the research question, or a dyadic chart. Following this, the dyadic charts were analysed for similarities and differences within and between the dyads.

In this secondary analysis, all existing interview data were included (rather than limiting it to only patients and partners in the pre-dialysis phase) as the participants on dialysis reflected on their experiences of starting dialysis, offering valuable perspectives which informed the research question. The interview transcripts and dyadic charts were re-read by CM to re-familiarise herself with the data. Then, the working themes were analysed by group, rather than by dyad, to allow differences in patients’ and partners’ experiences to emerge. The key themes for each group were then compiled using mind-maps to draw out their relations to QOL. These documents were then compared for similarities and differences between the themes. Next, the themes were integrated and refined to capture the experiences of both patients and partners. The research team continually reviewed the developing themes and examined how they related to patients’ and partners’ QOL. The themes were refined until everyone agreed they reflected the participants’ experiences.

#### Item generation

Preliminary items were drafted to address the concepts within each theme and drawn from the data to capture patients’ and partners’ language (see Additional file 1). Existing questionnaires related to these themes were re-assessed during item generation. An iterative process then commenced whereby the research team reviewed the draft version of the items and suggested that items be changed or removed. Changes were made to improve comprehensibility (e.g., making question structure clearer and reducing ambiguity) and add context (e.g., frame of reference such as ‘in 6 weeks’). Items were removed if they seemed repetitive or were difficult to understand. Response scales were then applied to the items, and further modifications were made to ensure consistency between question wording and response options.

### Phase 2

#### Design

Cognitive interviews were conducted from June to August 2017 with a subset of the participants from the qualitative semi-structured interview study (previously described in Phase 1), each participant being interviewed once.

Cognitive interviewing was selected as the method to examine the comprehensibility and applicability of the developing measure. In this qualitative method participants from the target population provide feedback on all aspects of the measure [8, 9]. Both think-aloud interviewing and concurrent verbal probes were utilised. In think-aloud interviewing, participants verbalise their thoughts about the question and how they form their responses. Concurrent verbal probes are prompts asked by the researcher to the participant immediately after a question has been answered which aim to explore the participant’s understanding of the question [8]. While think-aloud interviewing limits researcher bias, some participants may not be comfortable with this task or may speak about aspects of the items not immediately relevant to the research objectives [9]. To limit inadvertent bias, CM, who conducted the cognitive interviews, received training from an expert in instrument design prior to data collection.

#### Participants

All patients and their partners who took part in the over-arching study, and who gave their consent to be contacted in follow-up studies, were sent a letter of invitation and participant information sheet.

#### Procedure

The cognitive interviews were conducted at a time and location which was most convenient to the participants; four dyads chose to be interviewed in their home, and one dyad chose to be interviewed in a private room at the renal unit. All the cognitive interviews were conducted on an individual basis, except for one dyad who requested a joint cognitive interview.

Before starting the interview, CM introduced the SDQ, provided background on its development, its intended use in research, demonstrated how a cognitive interview was conducted and answered any questions about the study. The participants were given the relevant version of the SDQ and then asked to think out loud as they worked through each item. The participants were informed that CM may ask additional questions between items. The procedures and verbal probes for the cognitive interviews are provided in Table 1. All cognitive interviews were audio-recorded, and field notes documented after each interview. Interviews lasted an average of 67 min (range 33–101 min). Issues raised by a participant were then reviewed with the research team and discussed in subsequent cognitive interviews with other participants. The cognitive interviews concluded when participants raised no new issues. In the Results section, quotations are provided to elucidate key points made by the participants. To protect their confidentiality, the quotations are presented with limited identifiers (i.e., only patient or partner labels).

**Table 1:** Cognitive interview procedure and verbal probes

Introduction	The SDQ is going to be answered by people about to start dialysis The questions come from what we learned from the interview study you previously took part in. Today you are helping us make sure it makes sense and is understandable. This is a follow-up study called a cognitive interview, or think aloud task. It is different from the previous interview you took part in.
Demonstration of the task	*The researcher shows the participant the SDQ* *Then, the researcher provides a demonstration of thinking aloud using the following question (not on the SDQ):* *How would you rate your quality of life? 1* = *Very poor, 2* = *Poor, 3* = *Neither good nor poor, 4* = *Good, 5* = *Very good* *Researcher questions the time frame, what is meant by ‘quality of life’ and how she decides which response option to choose*
Instructions	Please complete the questionnaire, but as you work through each question please read it aloud and say what you are thinking. After you have answered a question, I may ask you questions about how you came to your answer. I will answer any question about the questionnaire once we are finished. Please know that you are not being tested—the questionnaire is being tested. There are no wrong or right answers.
Verbal probes	What does the term X mean to you? Can you repeat that question in your own words? How sure are you of your answer? How did you come to your answer? (What did you think about?) Was that easy or hard to answer? How do you feel about answering that question?

Cognitive interview procedures and verbal probes adapted from “Think-aloud, verbal probing, and other techniques” in [8], pp. 42–65

#### Analysis

Charts were created to collate the cognitive interview data, with separate charts for patients and partners. In the chart, the items of the SDQ formed the rows with each participant having a separate column. While listening to the audio-recordings, CM transcribed participants’ responses and added them to the appropriate chart. Field notes which offered additional context were included on the chart. Then, the responses were coded using a coding framework [10]. These were then compiled onto an overall chart to show the changes and the development of measure. The research team, with expertise in chronic illness, nephrology and questionnaire design, reviewed the outcomes of the cognitive interviews and approved of the changes made to the items.

### Phase 3

#### Study design and setting

Data obtained at baseline (pre-dialysis) in a longitudinal study [11] were used to evaluate the performance of the individual items, internal consistency, item correlations and relatedness of the domains. This study was conducted in 10 renal research units across England. Participants completed paper versions of the self-report questionnaires in their homes (95%) or in the renal clinic (5%); of these 97% completed the questionnaires without assistance.

#### Participants

Participants were recruited from the renal registries of the nephrology units from November 2017 to September 2018. The inclusion and exclusion criteria have been previously reported [11]. Briefly, patients were eligible to participate if: (1) they were in the care of a nephrologist for ESRD, (2) their clinical factors indicated that they were likely to start dialysis in the next 2 months, (3) they were planning to receive a form of out-patient HD or PD for the management of ESRD, (4) they were in a spousal-type relationship with someone they considered their “partner”, and 5) they were 18 years or older. Incident patients (i.e., those who had not been on a form of renal replacement therapy before) and patients who had a failing transplant and were planning to start dialysis (also referred to as prevalent patients in renal services), but had not been on any form of out-patient dialysis in the last 6 months, were included. Patients were not eligible for the study if they had acute kidney injuries or were receiving long-term inpatient dialysis for other health conditions. A partner was defined as a person in a spousal-type relationship who provided informal care in the form of emotional, physical and/or treatment-related support to an eligible ESRD patient [12]. Both patients and partners needed to be able to read and comprehend English.

#### Measures

The SDQ assesses patients’ and partners’ own views about starting dialysis across three conceptual domains (expectations of dialysis, accepting dialysis and dyadic relationship characteristics, DRC). There are two versions of the SDQ, one for patients and one for partners, and has been adapted for use at pre-dialysis and after starting dialysis. The analysis of preliminary psychometrics was conducted using data drawn from pre-dialysis measures only. In the pre-dialysis SDQ, the patient version consists of 33 items, and the partner version has 34 items. The questions use a 1 to 5 response scale and a response box for the two open-ended questions. The domains are scored separately and by calculating the mean of the items within each domain (scoring range 1–5), with high scores indicating high expectations that dialysis will improve health or QOL, being more accepting of dialysis and cohesive dyadic relationship characteristics. Fives items are reversed scored (accepting dialysis: 12, 13; DRC: 19, 30, 31). The domains contain the following number of items: expectations of dialysis (n = 7), accepting dialysis (n = 7) and DRC (patients: n = 17, partners: n = 18). Although the items within the measure address similar core concepts in both patient and partner versions, the phrasing differs slightly between them; therefore, the scores were examined separately by group.

Quality of life was assessed using the WHOQOL-BREF [13]. This instrument reflects a multi-dimensional model of subjective QOL in health and is assessed by 26 items. Two items form the WHOQOL general QOL facet and 24 specific items are scored in one of four domains: physical, psychological, social relationship and environment. The primary outcome variable in this phase of the study was WHOQOL general QOL, which is the mean of the overall QOL item (How would you rate your quality of life? 1 = Very poor, 2 = Poor, 3 = Neither good nor poor, 4 = Good, 5 = Very good) and the health-related QOL item (How satisfied are you with your health? 1 = Very dissatisfied, 2 = Dissatisfied, 3 = Neither satisfied or dissatisfied, 4 = Satisfied, 5 = Very satisfied) with a scoring range of 1–5. A score less than 3 is commonly regarded as indicating poor or very poor QOL whereas scores more than 3 suggest good to very good QOL.

Socio-demographic information (gender, age, relationship status, ethnicity, employment status, education) was collected via a self-report section in the questionnaires. Dialysis characteristics and clinical data were collected from patients’ medical records.

#### Analysis

Descriptive statistics (means, standard deviations, skew and kurtosis) were calculated for the individual items and the three domain scores by group. The skew and kurtosis values, as well as histograms for the individual items and domain scores, were inspected for non-normal distributions.

Cronbach’s α was used to examine the internal consistency of the domain scores and the contribution of each item to the domain. The internal consistency was deemed to be good if it was > 0.70 but < 0.90 [14]. Pairwise Pearson’s correlations were used to assess associations between all items, items and their proposed domain, and the relatedness of the domains to each other and QOL. Inter-item correlations were examined to identify redundant items (> 0.80) [14] and those that had high correlations with items in other domains. Items were interpreted as contributing to the domain if their item-domain correlations were > 0.40 [10]. Additionally, the correlations between the proposed domains were assessed to provide an indication of the relatedness of the constructs. Finally, the correlations between the proposed domains and WHOQOL general QOL were examined to assess their association with QOL.

## Results

### Phase 1

#### Participant characteristics

Of the 44 patients meeting the eligibility criteria, 22 patients (20 males and 2 females) were recruited with their partners. The reasons for non-recruitment were lack of response to the letter of invitation (n = 10), not interested in taking part (n = 7), responded after data collection was completed (n = 2), too busy (n = 2) and other reasons (n = 1). The characteristics of the participants are provided in Table 2.

**Table 2:** Characteristics of participants in Phase 1

	Patients (n = 22)	Partners (n = 22)
Male n (%)	20 (91%)	2 (9%)
Age *M* (range)	63 (39–80)	62 (39–87)
Married n (%)	20 (91%)	20 (91%)
*Ethnicity n (%)*		
White British	19 (85%)	18 (82%)
Asian	1 (5%)	2 (8%)
Black	1 (5%)	1 (5%)
Other	1 (5%)	1 (5%)
*Employment status n (%)*		
Retired	12 (54%)	12 (55%)
Unable to work	7 (32%)	3 (14%)
Full-time employment	3 (14%)	6 (26%)
Part-time employment	–	1 (5%)
*Phase of dialysis n (%)*		
Pre-dialysis	8 (33%)	–
Starting	7 (29%)	–
Establishing	7 (29%)	–
Mode of dialysis* n (%)		
Haemodialysis	16 (75%)	–
Peritoneal dialysis	6 (25%)	–

Phase of dialysis refers the point in the end stage renal disease (ESRD) trajectory the patients were in and defined as ‘pre-dialysis’ if receiving care for ESRD but not yet on a dialysis or a recipient of a renal transplant, ‘starting’ refers to patients on dialysis for < 6 months and ‘establishing’ refers to patients on dialysis for > 6 months but < 16 months

* Current mode of those on dialysis or intended mode of patients in the pre-dialysis phase

#### Qualitative analysis

Three themes, *dialysis expectations*, *accepting dialysis* and *dyadic relationship factors (DRC)*, were identified as relating to QOL in patients and partners in the early phases of dialysis. Table 3 provides an overview of the themes with a summary of the codes.

1. Dialysis expectations

**Table 3:** Themes and codes relating to QOL in patients and partners in early dialysis

*Dialysis expectations*	
Quality of life Health	Patients and partners spoke repeatedly about how their expectations had or had not been met by dialysis. Their primary concerns were on the impact dialysis would have on their QOL and the patient’s health
*Accepting dialysis*	
Treatment and illness Lifestyle Actively accepting and control of dialysis Future	Some patients and partners expressed the importance of accepting dialysis and the changes it brought to both of their lives. Some actively accepted dialysis whereas others seemed resigned to it. The amount of control they thought they had over dialysis and their view of dialysis as part of their future (i.e., whether they hoped for a transplant) factored into how accepting they were of dialysis
*Dyadic relationship characteristics*	
Team-like Communication Positivity Awareness of self and other	The analysis highlighted the importance of cohesive patient-partner relationships on their QOL. Those who worked as a team, communicated effectively, were positive, and showed an awareness for the other person seemed to be the least affected by dialysis

*QOL* quality of life

Participants discussed a range of expectations of dialysis, with some having very high expectations and others very low. Participants with high expectations which were not met by dialysis often reported their QOL as poorer than those who started dialysis with low expectations. Expectations centred around two key areas: the impact of dialysis on their QOL and the patient’s health. In regard to QOL, participants discussed their expectations of dialysis on their general QOL and several specific areas of QOL, such as: being able to do their day-to-day activities, ability to travel or have a holiday, restricted freedom and the impact on their social life. The other key area where expectations were formed was the patient’s health. Patients in particular talked about the expectations they had of dialysis on their energy and mobility, and they hoped that dialysis would allow them some ‘better’ days, which they discussed as important to improving their QOL.

2. Accepting dialysis

Across the phases of early dialysis, and in both patients and partners, participants who spoke of accepting dialysis discussed their QOL positively. Accepting dialysis occurred across different levels and included (1) the illness and treatment itself, (2) lifestyle changes as a consequence of it, (3) actively engaging with ESRD and dialysis and having some control and (4) the role of dialysis in their future. Those who accepted the routines and restrictions of dialysis and ESRD, often used social comparisons or reframing to downplay the impact of dialysis and facilitate acceptance. In regard to lifestyle, participants who stated they had a good QOL accepted the limitations imposed by dialysis and adjusted their lives to limit the negative effects of dialysis (e.g., finding hobbies that did not strain their energy). Those who actively accepted dialysis (as opposed to those who believed they had no choice about it) and those who talked about having control of dialysis spoke positively about their QOL. The final aspect of accepting dialysis was that participants who presented the best QOL had accepted dialysis as part of their future and were not pinning their hopes on a transplant. Those with high hopes for a transplant were less accepting of dialysis and were less able to minimise the impact of dialysis on their QOL.

3. Dyadic relationship characteristics

Couples, or dyads, that had good QOL had developed effective ways of communicating, working together, were mutually understanding and at least one of them was positive about dialysis. Dyads who adopted a team-like approach spoke about the importance of ‘being on the same page’, balancing their dialysis-related duties, each person’s involvement complementing the needs of the other, spending time together as a couple and being steadfast in their love and care for each other. The communication style within the dyad was important to how effectively and cohesively they worked together. It was important to be able to discuss fears, worries and issues about dialysis, but not let these overwhelm their lives and negatively impact their QOL. Being listened to, was noted as important. Both members of the dyads remarked that being positive and optimistic were critical to facing dialysis and maintaining their QOL. Cohesive dyads got strength from each other and used humour or normalised dialysis to promote positivity. These attributes were evident among those who stated their QOL was good. Being empathetic with each other and limiting the burdens on the other, characterised dyads who spoke of their QOL positively. Alongside being aware of each other, participants needed to be an individual within the dyad (i.e., getting time for one’s self or having a hobby outside of the dyad). In dyads where an awareness of each other was lacking, participants were more likely to cast blame on the other or talk about dialysis increasing their worry, loneliness or isolation.

#### Item generation

The next step in this phase was the generation of the items. The items in the SDQ ask about patients’ and partners’ *own* thoughts and feelings about dialysis expectations, accepting dialysis and DRC. During item generation existing questionnaires were reviewed and honed the conceptualisation of the theme ‘accepting dialysis’, which was originally labelled ‘acceptance’. A substantial body of research exists on acceptance, but this does not reflect the views expressed by the participants in our study, who discussed being accepting of dialysis as a treatment, rather than ESRD, the illness. In the DRC theme, four questions about communication were adapted from the Couples’ Illness Communication Scale [4] and used in the measure.

Two versions of the measure were required (one each for patients and partners). The items were adapted to be applicable to patients or their partners, and over two phases of dialysis (pre-dialysis and on dialysis). Across each version, the items addressed the same core concepts to facilitate comparisons between the patient and partner versions and over time. The pre-dialysis SDQs consisted of 33 questions on both patient and partner versions, and the dialysis SDQs had 34 questions. The items utilised 1–5 response scales adapted from the UK version of the WHOQOL [15]. The dialysis SDQs also contained two open-ended items with free text response boxes.

Both versions of the SDQ were reviewed by a patient and public renal research advisory group, consisting of five ESRD patients, a renal nurse, a lecturer and a professor in health services, who assessed its feasibility, acceptability and length of time to complete (approximately 20 min). They recommended using the term ‘partners’ rather than ‘caregivers’ and adding a question on relationship satisfaction. The SDQ was updated to reflect their feedback (see Additional file 2).

### Phase 2

#### Participant characteristics

Of the 22 couples invited, eight couples responded and five took part in the cognitive interviews. The reason three couples were not interviewed were due to (1) the patient dying before arranging the interview and his partner no longer wishing to take part, (2) unexpected poor health of a family member and (3) data collection having ceased when the couple responded. Participants’ characteristics are outlined in Table 4.

**Table 4:** Characteristics of participants in Phase 2

	Patients (n = 5)	Partners (n = 5)
Male n (%)	5 (100%)	0 (0%)
Age *M* (range)	62 (40–78)	58 (40–77)
Married n (%)	5 (100%)	5 (100%)
White British n (%)	4 (80%)	4 (80%)
*Employment status n (%)*		
Retired	4 (80%)	4 (80%)
Unable to work	1 (20%)	–
Full-time employment	–	1 (20%)
*Phase of dialysis n (%)*		
Pre-dialysis	1 (20%)	
Established	4 (80%)	
Length of time on dialysis—months (range)	21 (16–30)	–
Mode of dialysis* n (%)		
Haemodialysis	3 (60%)	–
Peritoneal dialysis	2 (40%)	–

Phase of dialysis refers the point in the end stage renal disease (ESRD) trajectory the patients were at and defined as ‘pre-dialysis’ if receiving care for ESRD but not yet on a dialysis or a recipient of a renal transplant and ‘established’ if the patient had been on dialysis for > 16 months

* Current mode of those on dialysis or intended mode of patients in the pre-dialysis phase

#### Results from the cognitive interviews

Overall, the concepts within the SDQ were found to be meaningful to the participants with one patient stating: “These are good questions to be asking people who may be experiencing these life issues for first time”.

The cognitive interviews revealed issues in comprehension, retrieval, judgement, responding and formatting (details provided in Table 5; Note: item identifiers refer to the question number on the patient – dialysis version of the developing SDQ). In total, eight of the questions did not require modification. Only five questions raised significant issues in their interpretation and comprehension, so were deleted (Q7, 8, 17, 27, 31). One question was found to be only applicable to partners (Q29) as it repeatedly needed clarification in meaning for patients (see Additional file 3 which provides a detailed account by item of the changes made and the development of the measure as a result of the cognitive interviews).

**Table 5:** Coding framework for cognitive interviews and frequency of issues

Aspects of information processing	Definition	Examples	Questions
*Comprehension*			
Difficult item	Delay in comprehending question meaning and difficulty answering	Q17: Dialysis affects patients’ lives in various ways and in early dialysis it is unlikely they could manage many tasks (2/10)	*Q7, 8*, 11, 12, *17*
Wording	Issue with wording or phrasing of the question	Q9: ‘Normal life’ discussed and ‘daily life’ suggested (3/10)	Q3, *7*, 9, 12, 15, 20, 32
Need for clarification	Participant needed more information to answer question	Q29: Patients asked if it included time on dialysis or just when they were off it (4/10)	Q1, 2, 29
Misinterpretation	Question not interpreted the way it was intended	Q21: Both patients and partners thought question asked about their own positivity (3/10)	Q21, 22, 29
Multiple interpretations	There are two or more possible interpretations	Q27: Talked about communication in regard to wider context rather than within couple and did not like phrasing (5/10)	Q10, 13, 16, *27, 31*
Semantic difficulties	The meaning of a word or phrase is not understood	Q1: Definition of QOL questioned and noted that everyone may define it differently (1/10)	Q1
Hesitation	Excessive pausing or re-reading while comprehending the question	Q26: Re-read question two times but confidently marked answer (1/10)	Q26
Incomprehension	The meaning of the question is not understood	NA	
Retrieval*			
Lack of information	Participants did not have knowledge that could inform question	Q3: Partners stated they had no or low expectations (3/10)	Q1, 3, 5, 16
*Judgement*			
Relevance	The extent to which the question is relevant to their experience	Q23: Partners commented that during early dialysis they did not express their feelings because they were focusing on being positive or still learning about dialysis (2/10)	Q1, 13, 14, 15, 21, 22, 23, 28
Repetition	A question has the same meaning as a previous one	Q7: Question similar to other Q9, Q16, Q17 (3/10)	*Q7, 8*, 11, 24, 25, 26
Time frame	Refers to the reference point for answering the question	Q1: Patient considered question in reference to QOL before chronic kidney disease rather than last 2 weeks (1/10)	Q1, 2, 9, 34
*Responding*			
Hesitation	Excessive pausing or hesitation	Q10: Partner thought about patient’s engagement in overall treatment (1/10)	Q3, 8, 10, 12, 25
Response scale confusion	Difficulty when marking response on the scale	Q22: Selected 1 when verbal reasoning indicated a score of 5 (1/10)	*Q7*, 22, 31, 33
Missed question	Question not answered, either intentionally or accidentally	Q13: Spoke about the question but could not select a response (1/10)	Q3, 12, 13
Response scale wording	Issues with wording of the response scale	Q32: Difficult to differentiate between 4 = A great deal and 5 = Completely (1/10)	Q1, *7*, 32
Response scale scoring	Issues with the scoring of the response scale	Q12: Noticed the scoring was different from previous questions (reverse scored) and recommended changing it to prevent mistakes (1/10)	Q2, 12, 25
*Other*			
Formatting	Changes to format recommended	Q9: Recommended moving it after Q6 (1/10)	Q4, 5, 9
*Positive feedback*			
Important question**	Question highlighted as important	Q6: Patient said it was key to adapting to dialysis (1/10)	Q6, 33, 34
Good question	Participant states that a question is good	Q12: Patient stated they liked it because it asked about an often-overlooked topic (1/10)	Q12, 20, 21, 22
Straight-forward question**	Participant states a question is straight-forward	Q11: Patients stated it was a clear question (2/10)	Q11

The question numbers match the item tested during the cognitive interviews (the developing versions of the Patient-Dialysis version of the Starting Dialysis Questionnaire, see Additional file 2). Adapted from [9]

*Retrieval was added to the framework and adapted from [8], p. 38

^**^Codes added by the research team

*Italic* question numbers indicate deleted questions

The cognitive interviews also underlined the extent to which partners are not prioritised during early dialysis. Partners repeatedly asked if the items were asking about their own thoughts, QOL and health. The enmeshment of the partners’ lives in the patients’ was made evident by one partner questioning if an item was about her own QOL and then forming her response by considering the patient’s health. Two partners stated that their thoughts and views were not important in early dialysis and that they held them back at that stage, “My views weren’t relevant at this point” and “I was afraid at first but still always holding something back”. In light of these comments, the instruction section in the final version of the partners’ questionnaires explicitly stated that we were interested in their own views and added emphasis to the word “your”, where appropriate.

In making judgements, participants often considered their response in reference to their lives before ESRD. To capture participants’ current thoughts, the instructions were altered to include the relevant time frame (e.g., the last 2 weeks).

On the basis of participants’ recommendations, items were added on expectations of dialysis at pre-dialysis, the impact on emotional health and feeling isolated due to dialysis. Modifications were made to the stems of items (e.g., non-leading openings such as ‘to what extent’ and ‘how much’) to facilitate their comprehensibility. Changes were also made in accordance with participants’ feedback on terminology (e.g., using the word ‘daily’ rather than ‘normal’ in Q9). Two items (Q16 and Q18) were moved because participants stated the topics aligned better with other items in the measure.

The end result of this phase of the study was the creation of patient and partner versions of the SDQ, with items adapted for use at pre-dialysis and after starting dialysis (see Additional file 4). The items address three domains, dialysis expectations, accepting dialysis and DRC. The patient versions consisted of 33 items at pre-dialysis and 30 items after starting dialysis. The partner versions comprised 34 items at pre-dialysis and 31 items after patients started dialysis. The readability of the questionnaire is at the basic level (Flesch-Kincaid grade level 5.5).

### Phase 3

#### Participant characteristics

Of the 153 patients invited to join the study, 83 couples (54%) consented and returned their questionnaires. Reasons given for not participating were: the patient or partner did not feel well enough (n = 12), too busy to take part (n = 8), patient started dialysis before informed consent and the first questionnaire were completed (n = 4), partner not interested in participating (n = 4), both members of the couple did not return their questionnaires (n = 2), patient died (n = 1), patient waiting to receive a transplant (n = 1), found the questions too intrusive (n = 2) and reason not offered (n = 36). Participants’ characteristics are provided in Table 6.

**Table 6:** Characteristics of participants in Phase 3

	Patients n = 83	Partners n = 83
*Socio-demographic characteristics*		
Male n (%)	52 (63%)	31 (37%)
Age *M* (*SD*, years)	64 (14)	63 (15)
Married n (%)	69 (84%)	70 (84%)
Highest level of education n (%)		
None	4 (5%)	4 (5%)
Primary school	3 (4%)	2 (2%)
Secondary school	40 (48%)	33 (40%)
College or training certification	25 (30%)	36 (43%)
University – undergraduate	4 (5%)	5 (6%)
University – postgraduate	6 (7%)	3 (4%)
Missing	1 (1%)	–
Ethnicity n (%)*		
White British	75 (91%)	77 (93%)
White Other	1 (1%)	1 (1%)
Asian Pakistani	2 (2%)	2 (2%)
Asian Other	3 (4%)	2 (2%)
Mixed/multiple ethnic groups	–	1 (1%)
Missing	2 (2%)	–
Employment status n (%)		
Retired	44 (53%)	45 (54%)
Working full-time	20 (24%)	15 (18%)
Working part-time	5 (6%)	10 (12%)
Unable to work	12 (14%)	6 (7%)
Do not work	–	6 (7%)
Missing	2 (2%)	1 (1%)
*Dialysis characteristics*		
Type of patient n (%)		
Incident patient	54 (65%)	–
Prevalent patient	6 (7%)	–
Missing	23 (28%)	–
Start of dialysis		
Planned	52 (63%)	–
Unplanned	4 (5%)	–
Missing	27 (32%)	–
Mode of dialysis n (%)		
HD	50 (60%)	–
PD	24 (29%)	–
Missing	9 (11%)	–
Type of access at pre-dialysis n (%)		
AVF	27 (33%)	–
Tesio line	7 (8%)	–
PD catheter	21 (25%)	–
Missing	28 (34%)	–
*Clinical variables*		
eGFR *M* (SD)	9.2 (3.3)	–
Haemoglobin g/L *M* (SD)	107.9 (15.9)	–
Serum albumin g/L *M* (SD)	37.9 (6.0)	–
Comorbidity risk n (%)		
Low	23 (28%)	–
Medium	42 (50%)	–
High	10 (12%)	–
Missing	8 (10%)	
Primary renal diagnosis n (%)		
Glomerulonephritis	10 (12%)	–
Polycystic	9 (11%)	–
Diabetes	7 (8%)	–
Renal vascular disease	5 (6%)	–
Hypertension	4 (5%)	–
Pyelonephritis	3 (4%)	–
Other	4 (5%)	–
Uncertain	7 (8%)	–
Missing	34 (41%)	

*AVF* arteriovenous fistula, *HD* haemodialysis, *PD* peritoneal dialysis, *eGFR* estimated glomerular filtration rate. Incident patient means a patient starting dialysis for the first time; prevalent refers to a patient who has been on a form of renal replacement therapy before but who intends to start dialysis due to a failing transplant

*Ethnicity codes taken from those used in UK renal units

#### Data examination

Questionnaire data was cleaned, and missing data assessed. Overall missing data by domain was as follows: dialysis expectations 3%, accepting dialysis 3% and DRC 2%.

#### Distribution of the responses

Table 7 provides the results of the preliminary evaluation of the psychometric properties of the patient and partner versions of the pre-dialysis SDQ by domain and item. The distribution of the responses indicated that participants used the full response scale with no evidence of ceiling or floor effects. There was no significant skew or kurtosis in the distribution of responses in any item (defined as values above 1.0 for each) with the highest skew in Q10 (patients) and Q28 (partners). On the basis of distribution, no items exhibited characteristics suggesting they should be modified or discarded.

**Table 7:** Psychometric properties of the pre-dialysis Starting Dialysis Questionnaire

		Patients (n = 83)							Partners (n = 83)						
		Mean	SD	Skew	Kurtosis	Item-domain correlation	Alpha (α) if item removed	Alpha (α)	Mean	SD	Skew	Kurtosis	Item-domain correlation	Alpha (α) if item removed	Alpha (α)
*Expectations*		3.35	0.67	0.31	0.23			0.90	3.17	0.48	0.00	0.00			.86
1.	In 6 weeks, what do you think your quality of life will be like?	3.29	0.69	0.09	0.45	0.77	0.88		3.06	0.50	0.63*	0.00	0.65	0.84	
2.	In 6 weeks, what do you think your *physical* *health* will be like?	3.27	0.71	0.42	0.87*	0.75	0.88		3.07	0.49	0.00	0.00	0.56	0.85	
3.	In 6 weeks, what do you think your *emotional health* will be like?	3.23	0.80	0.53*	0.37	0.72	0.88		3.02	0.54	0.06	0.01	0.79	0.82	
4.	In 12 weeks, what do you think your quality of life will be like?	3.43	1.02	0.75*	0.00	0.87	0.86		3.24	0.79	0.00	0.22	0.83	0.80	
5.	In 12 weeks, what do you think your *physical* *health* will be like?	3.35	0.99	0.62*	0.00	0.81	0.87		3.13	0.68	0.00	0.00	0.77	0.82	
6.	In 12 weeks, what do you think your *emotional health* will be like?	3.33	0.94	0.33	0.62*	0.78	0.88		3.15	0.80	0.00	0.06	0.78	0.81	
7.	How would you rate your expectations of dialysis?	3.48	0.69	0.07	0.74*	0.25~	0.93^		3.50	0.71	0.00	0.08	0.15~	0.90^	
*Accepting dialysis*		3.29	0.61	0.46	.05			0.75	3.41	0.67	0.22	0.66*			0.81
10.	How much have you come to terms with starting dialysis?	3.35	1.15	0.95*	0.04	0.62	0.68		3.78	1.04	0.28	0.01	0.70	0.77	
11.	To what extent do you think you will be able to carry on with your daily life when you start dialysis?	3.26	0.75	0.32	0.12	0.55	0.71		3.30	0.83	0.73*	0.21	0.49	0.80	
12.	How much do you think you will be bothered by dialysis?	3.05	0.87	0.01	0.62*	0.67	0.68		3.20	0.99	0.26	0.59*	0.48	0.80	
13.	How bothersome do you expect dialysis to be for your partner?	3.04	0.95	0.86*	0.84*	0.22~	0.77^		2.70	0.98	0.00	0.25	0.61	0.77	
14.	To what extent do you think you will have the control of dialysis that you would like?	3.14	1.02	0.82*	0.38	0.30~	0.76^		3.32	1.02	0.48	0.04	0.49	0.80	
15.	How satisfied are you that dialysis is the best option for you at this time?	4.02	0.79	0.00	0.01	0.43	0.73		4.28	0.69	0.02	0.49	0.44	0.80	
16.	How bothered would you be if dialysis became a long-term treatment for your kidney disease?	3.18	1.19	0.86*	0.02	0.55	0.70		3.28	1.25	0.79*	0.00	0.65	0.77	
*Dyadic relationship characteristics*		3.95	.06	0.03	0.58			0.92	3.85	0.59	0.08	0.64*			0.90
17.	How much do you expect that your partner will be involved in your dialysis?	3.58	1.03	0.48	0.03	0.57	0.91		3.93	0.89	0.13	0.14	0.46	0.90	
18.	How much do you think your partner’s involvement in your dialysis will match your needs?	3.89	0.94	0.01	0.62	0.62	0.91		3.94	0.97	0.02	0.87*	0.54	0.90	
19.	How much do you expect dialysis will change your role in the relationship?	3.78	1.09	0.05	0.20	0.13~	0.93^		3.76	1.11	0.03	0.19	0.34~	0.90	
20.	How much do you think you and your partner will act as a team when it comes to handling your dialysis?	4.16	0.87	.00	0.06	0.74	0.91		4.12	1.08	0.00	0.03	0.53	0.89	
21.	How much do you think that you and your partner will be “on the same page” (share similar views) about dialysis?	4.0	0.87	0.19	0.01	0.72	0.91		4.11	0.86	0.01	0.84*	0.57	0.90	
22.	How positive do you think you will be about dialysis?	3.88	0.85	0.61*	0.01	0.60	0.91		4.20	0.75	0.05	0.45	0.40~	0.90	
23.	How positive do you think your partner will be about dialysis?	3.94	0.93	0.17	0.01	0.66	0.91		3.78	0.94	0.17	0.06	0.58	0.90	
24.	How well do you think you will be able to express your *feelings* about dialysis to your partner?	4.12	1.00	0.00	0.79*	0.71	0.91		3.95	1.04	0.00	0.53*	0.68	0.89	
25.	How comfortable do you think you will be discussing *issues* related to dialysis with your partner?	4.30	0.87	0.00	0.36	0.70	0.91		4.24	0.90	0.00	0.07	0.69	0.89	
26.	How comfortable do you think your partner will be to talk about dialysis-related *issues*?	4.20	0.87	0.01	0.46	0.71	0.91		4.00	1.16	0.00	0.38	0.80	0.89	
27.	How willing do you think your partner will be to share his/her *feelings* about dialysis with you?	3.98	1.06	0.01	0.32	0.73	0.91		3.98	1.20	0.00	0.49	0.76	0.89	
28.	How much do you think that your partner will listen to your views on dialysis related topics?	4.20	0.88	0.01	0.31	0.74	0.91		3.88	0.86	.99*	0.00	0.49	0.90	
29.	*Partner only@@*How often do you think you will you get time for yourself once dialysis starts?	–	–	–	–	–	–		3.00	0.78	0.21	0.31	0.38~	0.90	
30.	How often do you think you will feel lonely because of dialysis?	3.80	0.81	0.07	0.17	0.56	0.91		3.60	1.05	0.20	0.24	0.57	0.90	
31.	How often do you think you will feel isolated because of dialysis?	3.70	0.91	0.08	0.88*	0.49	0.92		3.67	1.00	0.10	0.83*	0.51	0.90	
32.	How often do you think that you and your partner will do activities you enjoy together?	3.27	1.04	0.24	0.03	0.59	0.91		3.04	0.86	0.01	0.47	0.52	0.90	
33.	How often do you expect that you and your partner will be able to find humour in small things or have a laugh?	3.91	0.96	0.19	0.00	0.55	0.91		3.73	1.06	0.21	0.18	0.53	0.90	
34.	How satisfied are you with your relationship?	4.43	0.87	0.00	0.08	0.54	0.93^		4.38	0.73	0.00	0.56*	0.53	0.90	

The wording of the items presented here are taken from the pre-dialysis patient version of Starting Dialysis Questionnaire. Items on the pre-dialysis partner version differ slightly in phrasing (see Additional file 4)

*Item with minor skew or kurtosis, values > 0.5

^Item, that if removed, the internal consistency of the domain improves

^~^Item with low contribution to the domain, values < 0.40

#### Internal consistency

On initial assessment all the domains showed good internal consistency in both patient and partner versions. The items within each domain were evaluated using item substitution which provides α for the domain if the item is removed. The internal consistency of domains improved if some items were removed (patients: 5 items—Q7, Q13, Q14, Q19, Q34; partners: 1 item—Q7).

#### Item correlations

Correlations between each item and all other items in the domain and across the measure were examined to provide a preliminary assessment of the structure of the SDQ. Overall, the items correlated most highly with the other items in the proposed domains. However, in the patient version, four items had poor item-domain correlations (*r* < 0.40; Q7, Q13, Q14, Q19) and did not have strong correlations (*r* > 0.50) with any other item on the SDQ. Two items in the DRC domain had strong correlations with items in the accepting dialysis domain (Q22 with Q13, *r* = 0.55; Q31 with Q11, *r* = − 0.58). In the partner version, four items had weak item-domain correlations (Q7, Q19, Q22, Q29) and no strong correlations with any other items. Additionally, two items in the DRC domain had strong correlations with items in the accepting dialysis domain (Q23 with Q13, *r* = − 0.57; Q25 with Q10, *r* = 0.56) but had stronger correlations with the other items in the DRC.

#### Domain relatedness

The domain scores within patient and partner versions were assessed for relatedness between the constructs. In the patients, dialysis expectations and accepting dialysis were positively correlated (*r* = 0.32, *p* < 0.05) and accepting dialysis and DRC were also positively correlated (*r* = 0.44, *p* < 0.05). Expectations and DRC did not have a statistically significant correlation (*r* = 0.11, *p* > 0.05). In the partners, there was a significant positive correlation between accepting dialysis and DRC (*r* = 0.48, *p* < 0.05). The other domains were not significantly correlated (dialysis expectations and accepting dialysis, *r* = 0.09, *p* > 0.05; dialysis expectations and DRC, *r* = 0.10, *p* > 0.05). These results suggest that the domains are separable constructs which are weakly to moderately related to each other.

The domain scores in patients and partners were assessed for correlations with the WHOQOL general QOL scores. In patients, the dialysis expectations domain was negatively correlated with general QOL (*r* = − 0.27, *p* < 0.05) but neither accepting dialysis nor DRC, as measured here, were correlated with general QOL (*r* = 0.02, *p* > 0.05; *r* = 0.12, *p* > 0.05). In partners, DRC had a significant positive correlation with QOL (*r* = 0.23, *p* < 0.05) but dialysis expectations and accepting dialysis were not correlated with QOL (*r* = 0.4, *p* > 0.05; *r* = 0.17, *p* > 0.05).

#### Recommendations

The results of the preliminary psychometric analysis showed that some items in each version of the SDQ did not perform as well as the others. In the dialysis expectations domain, Q7 had some kurtosis, low correlation with other items and did not contribute to the internal consistency. Only in patients were there items (Q13 and Q14) in the accepting dialysis domain that were problematic across the assessments. In the DRC domain, Q19 (patient and partners), Q22 (patients and partners), Q29 (partners only) and Q31 (patients) did not perform as well as other items; however, these were not across all assessments (e.g., distribution, internal consistency, item correlations). In the patient versions, high correlations between Q22 and Q31 with items in the accepting dialysis domain may suggest these items should be removed from the DRC domain and considered part of the accepting dialysis domain. Although Q23 and Q25 had strong correlations with items in the accepting dialysis domain, overall they correlated more highly with items in the DRC domain and, therefore, should not be moved to the accepting dialysis domain.

Changes to the domains on the basis of these recommendations improved the internal consistency of the dialysis expectations domain (patients: α = 0.93, partners: α = 0.90), accepting dialysis domain (patients: α = 0.84, partners: not applicable).However, the internal consistency of the DRC domain only improved slightly for partners (α = 0.90) and decreased for patients (α = 0.90). Future versions of the SDQ should consider the removal of Q7 (both versions), Q13 and Q14 (patient versions) and should re-evaluate the performance of Q19, Q22 (both versions), Q29 (partners) and Q31 (patients).

## Discussion

In this study we aimed to develop a measure which assesses key psychological and interpersonal factors in patients and partners during the early phases of dialysis. Using qualitative methods, we identified three factors, namely dialysis expectations, accepting dialysis and DRC, as important during this time period. Then we generated questions, derived from rich interview data, which addressed these constructs. Next, we employed cognitive interview techniques to evaluate the comprehensibility and suitability of the questions in a sample of patients with ESRD and their partners. Finally, preliminary psychometric analyses were conducted on both patient and partner versions of the pre-dialysis SDQ and indicated that overall, it has good individual item performance and internal consistency. The emerging evidence in regard to the interrelatedness of the domains suggests that they are distinct yet may be related to each other and differentially to QOL. The SDQ is a person-centred measure which may prove to be a useful tool to assess psychological and dyadic factors in patients preparing to start dialysis and their partners.

The development of the SDQ arose after a review of existing questionnaires in the chronic illness and ESRD literature found none that could be adapted to address the specific experience of starting dialysis and which were applicable to patients’ partners. While repositories provide access to validated measures, and items across a range of illnesses and populations (e.g., Patient-Reported Outcomes Measurement Information System), the usage of these would not have met the nuanced experiences of couples preparing to start dialysis. Secondary analysis of existing in-depth qualitative data provided an opportunity to fill this gap by creating a measure which addresses important factors that impact patients *and* their partners during this crucial time in dialysis.

The finding that DRC is important during the early phases of dialysis, and also related to accepting dialysis and partners’ QOL, complements findings reported in qualitative research with ESRD dyads. Wise, Schatell, Klicko, Burdan, and Showers [16], who conducted interviews with patients who had started on short daily home HD (SDHHD) and their partners, noted the importance of relationship characteristics in how dyads’ adjusted to SDHHD. Dyads who were adjusted well were optimistic, in a solid relationship, shared duties, had clear roles, communicated effectively and were mutually respectful. Similarly, patients who had recently started HD and their family members described the significant impact dialysis had on their roles and personal relationships [17]. Taken together, these findings highlight the vital role the dyadic relationship plays in accepting dialysis and influencing QOL, especially in partners.

There is limited research which has explored the role expectations play in QOL over the transition onto dialysis. Our qualitative findings indicated that high expectations of dialysis, which if not met, are associated with poor QOL when the patient starts dialysis. This relationship between dialysis expectations and QOL was supported by the preliminary psychometric evaluation where patients’ high dialysis expectations were negatively associated with their QOL. This echoes the findings of Stringer and Baharani [18] who found patients’ high expectations of dialysis diminished markedly after starting. Further research is needed to determine the role of dialysis expectations on QOL in both patients and their partners.

Accepting dialysis is a different perspective on acceptance as it focuses on the treatment rather than the illness. In our analysis of the qualitative interviews, we identified that accepting dialysis had sub-levels comprising both cognitive and behavioural elements. These findings complement a review by Chan [19] who differentiated between stoic and active acceptance. Active acceptance involved mental processes, such as reconciling negative impact of the illness, and engaging in lifestyle changes. The accepting dialysis domain in the SDQ may provide clinicians a quick and useful way to profile patients and partners and assess their style of acceptance. Although we did not find the relationship between being accepting of dialysis and QOL to be strongly correlated, other research with dialysis patients provides evidence that accommodative coping styles, in which acceptance is a key feature, are positively associated with QOL [20].

The domains of the SDQ complement the biopsychosocial model of QOL which found cognitive appraisals (e.g., illness perceptions, control of illness) and social support to have a medium effect on dialysis patients’ QOL [21]. This model of QOL and its psychosocial correlates has not yet been explored in the partners of dialysis patients, and the evidence in this present study suggests DRC, a specific element of social support (i.e., the patient-partner dyad), is related to QOL in partners. Although research with partners of ESRD patients is burgeoning, there remains little consensus as what to measure and how to measure it [22]. A majority of research examines burden and affect with limited consideration of partners’ cognitive appraisals or interpersonal relationship with the patient. Furthermore, these studies tend to be conducted once patients are established on dialysis rather than during the early phases of dialysis.

As little is known about the experiences of patient-partner dyads in the early phases of dialysis, our usage of existing semi-structured interview data permitted an inductively driven approach to identify constructs and to generate questions. In Phase 2, the cognitive interviews further enhanced the measure by ensuring the items used language that was accessible and comprehensible. Through the cognitive interviews, valuable insight was gained into complex issues such as item interpretation and response judgement (i.e., partners revealed the extent to which their views are not sought; patients often recalled healthy periods in their life rather than their recent health, hence the need to set clear time frames within the measure). While the researcher aimed to conduct all the cognitive interviews individually, one couple requested their cognitive interview be conducted together. Although shorter in duration than the other cognitive interviews, the findings proved insightful as they debated question meanings and terms between themselves yielding rich natural data that occurs in focus groups.

A limitation of this study was that factor analyses were not conducted to assess the structure of the SDQ due the limited sample sizes in each group. Large field trial data, analysed with advanced multivariate techniques, will be necessary to establish the measure’s final structure and complete validation (e.g., construct, convergent, divergent) before it can be used in a clinical setting.

Another potential limitation is that the participants in the cognitive interviews also took part in the over-arching qualitative study and were predominantly White British male patients with female spouses. It is possible that their experiences and the psychosocial factors that impacted them may not be reflective of the experiences of female patients with ESRD or male partners or the experiences of non-White patients and partners from diverse cultures. The renal research advisory group, which included a female patient, reviewed the initial draft of the SDQ and confirmed the relevancy of the questions. Another consideration is that four of the couples had been on dialysis for more than one year when they took part in the cognitive interviews. Being established on dialysis may have affected their ability to recall their own attitudes about starting dialysis or identify additional factors important during the early weeks. A strength of this measure is that it reflects topics that patients and their partners stated as important to them to during a stressful time in ESRD treatment and, thus, is a patient-reported outcome measure [23]. Furthermore, participants had the opportunity to comment on items which were derived from a data set to which they contributed. Their participation in the semi-structured interviews may have facilitated their ability to engage in the cognitive interviews (which can be a difficult research task for some people) because they were familiar with the topic and the researcher.

## Conclusion

The SDQ is a brief measure which assesses key psychological and interpersonal factors that are important to patients and their partners as they prepare to start dialysis. It comprises three conceptual domains which were identified using qualitative methods. To ensure its relevance to patients and partners, the items were created using a data-driven approach and then assessed for comprehensibility through cognitive interviews. The preliminary psychometric evaluations indicate that the items and domains perform well, and there is emerging evidence that they are related to QOL. The SDQ may offer clinicians a practical tool to identify patient-partner dyads who would benefit from additional support or counselling as they prepare to start dialysis.

## Supplementary information

**Additional file 1**. Initial questions by theme and code.

**Additional file 2**. Developing versions of the Starting Dialysis Questionnaire.

**Additional file 3**. Chart showing original questions assessed in cognitive interviews, actions and final questions.

**Additional file 4**. Final pre-dialysis versions (patient and partner) of the Starting Dialysis Questionnaire.

## Data Availability

The dataset supporting the conclusions of this article are available from the corresponding author on reasonable request.

## Abbreviations

eGFR: Estimated glomerular filtration rate
DRC: Dyadic relationship characteristics
ESRD: End stage renal disease
HD: Haemodialysis
PD: Peritoneal dialysis
QOL: Quality of life
SDQ: Starting Dialysis Questionnaire
WHOQOL-BREF: World Health Organization Quality of Life 26-item questionnaire

## References

[1] Jablonski A (2004). The illness trajectory of end-stage renal disease dialysis patients. Res Theory Nurs Pract..

[2] Revenson TA, Griva K, Luszczynska A, Morrison V, Panagopoulou E, Vilchinsky N (2016). Caregiving as a dyadic process. Caregiving in the illness context.

[3] Moore C, Skevington S, Wearden A, Mitra S (2019). Impact of dialysis on the dyadic relationship between male patients and their female partners. Qual Health Res.

[4] Arden-Close E, Moss-Morris R, Dennison L, Bayne L, Gidron Y (2010). The Couples' illness communication scale (CICS): Development and evaluation of a brief measure assessing illness-related couple communication. Br J Health Psychol.

[5] Braun V, Clarke V (2006). Using thematic analysis in psychology. Qual Res Psychol..

[6] Braun V, Clarke V (2013). Successful qualitative research: a practical guide for beginners.

[7] Eisikovits Z, Koren C (2010). Approaches to and outcomes of dyadic interview analysis. Qual Health Res.

[8] Willis GB (2005). Cognitive interviewing: A tool for improving questionnaire design.

[9] Willis GB, Artino AR (2013). What do our respondents think we're asking? Using cognitive interviewing to improve medical education surveys. J Grad Med Educ.

[10] Mason VL, Skevington SM, Osborn M (2008). The quality of life of people in chronic pain: Developing a pain and discomfort module for use with the WHOQOL. Psychol Health..

[11] Moore C, Carter L-A, Mitra S, Skevington S, Wearden A (2020). Quality of life improved for patients after starting dialysis but is impaired, initially, for their partners: a multi-centre, longitudinal study. BMC Nephrol..

[12] Revenson TA, Griva K, Luszczynska A, Morrison V, Panagopoulou E, Vilchinsky N (2016). What is caregiving and how should we study it? Caregiving in the illness context.

[13] Skevington SM, Lotfy M, O'Connell K (2004). The World Health Organization's WHOQOL-BREF quality of life assessment: Psychometric properties and results of the international field trial—a report from the WHOQOL group. Qual Life Res.

[14] Streiner DL, Norman GR (2008). Health measurement scales: a practical guide to their development and use.

[15] Skevington SM, Tucker C (1999). Designing response scales for cross-cultural use in health care: data from the development of the UK WHOQOL. Br J Med Psychol.

[16] Wise M, Schatell D, Klicko K, Burdan A, Showers M (2010). Successful daily home hemodialysis patient-care partner dyads: benefits outweigh burdens. Hemodial Int.

[17] Monaro S, Stewart G, Gullick J (2014). A 'lost life': coming to terms with haemodialysis. J Clin Nurs.

[18] Stringer S, Baharani J (2012). Why did I start dialysis? A qualitative study on views and expectations from an elderly cohort of patients with end-stage renal failure starting haemodialysis in the United Kingdom. Int Urol Nephrol.

[19] Chan R (2012). The effect of acceptance on health outcomes in patients with chronic kidney disease. Nephrol Dial Transplant.

[20] Poppe C, Crombez G, Hanoulle I, Vogelaers D, Petrovic M (2013). Improving quality of life in patients with chronic kidney disease: influence of acceptance and personality. Nephrol Dial Transplant.

[21] Chan R, Brooks R, Steel Z, Heung T, Erlich J, Chow J (2012). The psychosocial correlates of quality of life in the dialysis population: a systematic review and meta-regression analysis. Qual Life Res.

[22] Gilbertson EL, Krishnasamy R, Foote C, Kennard AL, Jardine MJ, Gray NA (2019). Burden of care and quality of life among caregivers for adults receiving maintenance dialysis: a systematic review. Am J Kidney Dis.

[23] Weldring T, Smith SMS (2013). Patient-reported outcomes (PROs) and patient-reported outcome measures (PROMs). Health Serv Insights.

