# Supplementary material for: Development of a measure for patients preparing to start dialysis and their partners: The Starting Dialysis Questionnaire (SDQ)
Source: Health Qual Life Outcomes. 2020 Nov 7;18:358. doi: 10.1186/s12955-020-01610-x (PMC7648298; doi:10.1186/s12955-020-01610-x)
Supplement: Supplementary file 1 — Additional file 1. Initial questions by theme and code. [file 12955_2020_1610_MOESM1_ESM.docx]

| Additional file 1: *Initial questions by theme and code* | | |
| --- | --- | --- |
| *Theme and code* | Example quotation | Questions |
| *Dialysis Expectations* |  |  |
| Quality of life | *“Hopeful it will improve my quality of life.” (Patient)* | What kind of effect do you think dialysis will have on your QOL? |
| Able to do day-to-day activities | *“Normally I just go and get a bulb and fix it, climb up the ladder and do it. I found I couldn’t do that. And also things like that are very depressing.” (Patient)*  *“We're able, we make sure that we go out, it may just be a little walk around the shops, it might be for lunch but we try to make non-dialysis days normal days where we can.” (Partner)* | *How satisfied are you with your ability to get out of the house and do the things you want to do? |
| Travel & Holidays | *“It's so important to have that quality of life so you can actually go for your holidays or you can just enjoy it.” (Partner)* | *How concerned are you about the effect dialysis will have on your ability to travel or have holidays? |
| Restricted freedom | *“I feel fitter four days a week because I have dialysis three days a week, so the day afterwards I walk a bit quicker, I've got a bit more life in me than I used to have. Having said that it's all consuming, it's all hospitals and tying yourself up to a machine. You’ve always got to think, oh I can't do that on a Tuesday, and I can't do that – so it is life altering.” (Patient*) | *How concerned are you that dialysis will restrict your freedom? |
| Social life | *“So at least now he can go out when he wants. But he’s not been going out, but it will start again.” (Partner)* | *How concerned are you about the effect dialysis will have on your social life? |
| Health | *“So I think really just probably worried about what’s going to happen and worried about how long it might take until a kidney’s found, and then sort of thinking, well, hopefully when he’s had it done he’s going to feel much better.” (Partner)* | What do you think your health will be like 6 weeks from now? |
| *Accepting dialysis* |  |  |
| Dialysis |  |  |
| Dialysis | *“There again that’s [dialysis] going to alter your life, but you have to accept that, you know. No, you don’t have to, but we would. I would, I’ve accepted it. That’s our life for the next three months (Partner)* | How able are you to accept dialysis? |
| ESRD | *“There's not a lot I can do about my body. I try to, you know, maintain a bit of a healthy diet but you’ve just got to get on with it, yeah.” (Patient)* | *Have you come to terms with ESRD? |
| Regime/routine of dialysis | *“But, as I say, you just get a new routine, don’t you, because there's nothing else you can do. But you just get in a routine, don’t you? You’ve got to get on with it, what else can you do?. . . You’ve got no option, have we.” (Partner)* | How accepting are you of the routine and amount of time spent on dialysis? |
| Context | *“I think because we’re older as well, I think as you get older you just – well, you’re together and you love one another and illnesses and things happen, don’t they, that are always there to test you.” (Partner)* | *How have previous medical experiences influenced your ability to accept dialysis? |
| Lifestyle |  |  |
| Limitations | *“We can't do that anymore, you know, we have to go to places that it's safe to go to with [patient]. But, I've been looking into other, sort of, holidays and you know, as long as we can get away, you know, it's not a huge disappointment that really.” (Partner)* | How able are you to accept the limitations of you/your partner due to dialysis? |
| Adjustment | *“You just accept, well at some stage it’s going to happen.” (Patient)* | How much are you able to accept the changes to your life that dialysis may bring? |
| Adaptation | *“I'm hoping that we'll be able to fit dialysis around our life as opposed to the other way around.” (Partner)* | *How much are you able to accept dialysis because you are confident you can adapt dialysis to your life? |
| Actively accepting & control of dialysis |  |  |
| Difficulties accepting | *“I never wanted to have dialysis but I'm going to have no choice, by the look of it. . . I'm not looking forward to dialysis.” (Patient)* | How hard have you found it to accept dialysis as part of your life? |
| Lack of choice | *“What can you do, you’re stuck. . . Because there’s no choice.” (Partner)* | To what extent do you accept dialysis as part of your life because you feel you had no other choice? |
| Benefits | *“You must… it’s burden or no burden, but you must do it to keep alive. There’s no way out. If you don’t do it you’ll go downhill, it’s that straightforward.”(Patient)* | To what extent do you accept dialysis because it will lead to benefits for you or your partner (health, positive future)? |
| Ownership/Control | *“But it is fantastic when the option is there, that you can have it at home and you can kind of control your own disease. . . And you taking over it, not that your disease is taking over you.” (Partner)* | Does your dialysis give you the ownership and control of your life that you would like? |
| Future |  |  |
| Transplant | *“I try and make the best of it in the hope that I'm going to get a kidney. Because I reckon that if I get a kidney and it's all alright, I should get some of my life back then.” (Patient)* | How hopeful are you that you/your partner will receive a transplant in the near future? |
| *Dyadic Factors* |  |  |
| Team-like |  |  |
| Togetherness | *“I mean emotionally we’ve always been there together” (Patient)* | To what extent do you feel like you and your partner are a team when it comes to handling your dialysis? |
| Steadfastness | *“Because I decide I’m married to her and whatever happens, whether good or bad, I’ll be there for her 100 plus percent, no matter what.” (Partner)* | *How much do you and your partner share the belief that no matter what happens you will do your best to look after each other? |
| Role in Relationship | *“I’m one of these people that do everything. You know, I do all the house, the gardening and my husband works and so I always did everything. . .So it’s not fair really, I don’t think, that he should have to do it, but he does.” (Patient)* | To what extent has dialysis changed your role in the relationship? |
| Role in dialysis | *“I think that changes, because you become a carer, not his partner. I’m his carer.” (Partner)* | How content are you with the roles each of your have in relation to dialysis? |
| Being a couple | *“Sometimes you’ll see couples out doing stuff and it’s always me on my own just doing everything by myself and.. I think it doesn’t get to me all the time but sometimes I just think, ‘oh, wouldn’t it be nice if we could share these things?’” (Partner)* | How important to you is it that you and your partner make time to do things as couple? |
| Communication |  |  |
| Communication style | *"I've been told to communicate quite a lot." (Patient)* | How would you describe your communication with your partner about dialysis |
| Fears | *“It’s the fear factor of that next level of…well, he’s stable now. We’re going to upset the apple cart. What’s going to happen, what’s…it’s the fear factor.” (Partner)* | To what extent are you able to share your fears about dialysis or the future with your partner? |
| Worry | *“[Whispers to CM] I don’t let him know that I worry. Don’t let him see me worried.” (Partner)* | How often do you limit what you tell your partner about dialysis to prevent her/him from worrying? |
| Listening | *“And taking on board what I say. I think before he used to not listen. ‘Cause it’s been like all the things you say, you say something and it works for a few days or weeks and then it all drifts back to how it was before.” (Partner)* | Does your partner listen to you and take your point of view on board? |
| Positivity |  |  |
| Positive | *“I'm trying to twist things for him to make it look more positive. As I say, you know, you're only doing three days, you're not doing four, and you can still go out of a morning.” (Partner)* | How much does your partner rely on you to feel positive about dialysis? |
| Strength | *“And, as I said, [patient] is very strong and I'm able to leech his strength and together we get through it.” (Partner)* | How much do you rely on your partner for strength or positivity? |
| Humour | *Yeah, because we’ve been through so much and we can laugh about it. (Partner)* | How often are you and your partner able to find humour in small things and have a laugh? |
| Awareness of self and other |  |  |
| Pressure | *“There were times that I felt out of my depth and I wasn’t comfortable. I was out of my comfort zone, looking after him.” (Partner)* | *How often you feel overwhelmed by the pressure to manage dialysis related tasks as well as your other everyday tasks? |
| Burden | *“It's like having another baby for her, having me.” (Patient)* | How much of a burden is dialysis on you?  How much of a burden is dialysis on your partner? |
| Burden | *“When I’ve got energy...I can do my fair share of the jobs that need to be done. . . [but I’m not satisfied because] I’m not delivering my share of the bargain.” (Patient)* | *How much do you worry that you are a burden to your partner because of dialysis? |
| Loneliness/Isolation | *“If the girls didn’t come, you know, I’d be quite honest it’s a bit lonely.”*  *(Partner)* | How has dialysis effected the level of loneliness or isolation you feel? |
| Time for self | *“I can relax. So, yeah, I make cards and I knit and I’ve got into doing word searches. I just find that is a chilling out thing, sort of thing.” (Partner)* | How often are you able to get the time you need for yourself? |
| * Denotes questions cut during the refinement process of developing the preliminary versions of the questionnaire. Items were removed because they were similar to other questions, could not be simplified to address a core concepts or replicated items on other scales that formed part of Phase 3. | | |
