# Supplementary material for: Development of a measure for patients preparing to start dialysis and their partners: The Starting Dialysis Questionnaire (SDQ)
Source: Health Qual Life Outcomes. 2020 Nov 7;18:358. doi: 10.1186/s12955-020-01610-x (PMC7648298; doi:10.1186/s12955-020-01610-x)

Additional file 2: Developing versions of the Starting Dialysis Questionnaire (originally referred to as the Psychosocial Factors Questionnaire)

*SDQ – Pre-dialysis – Patient version*


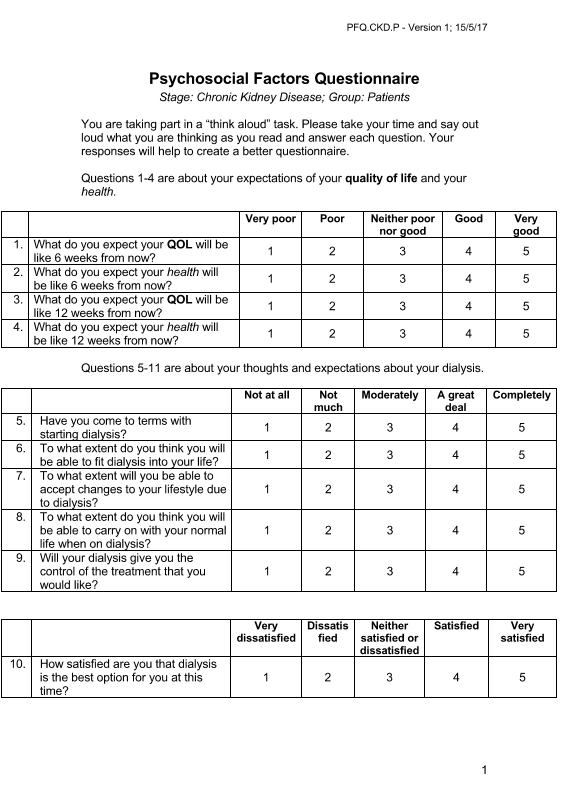


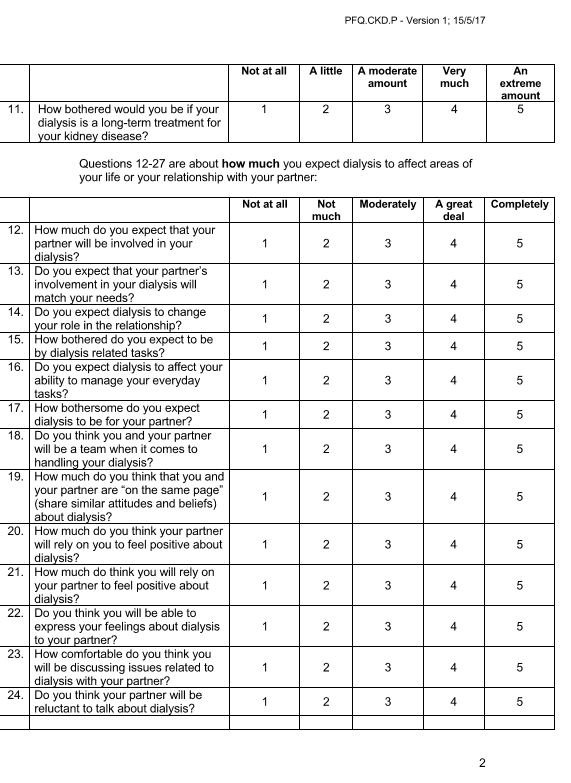


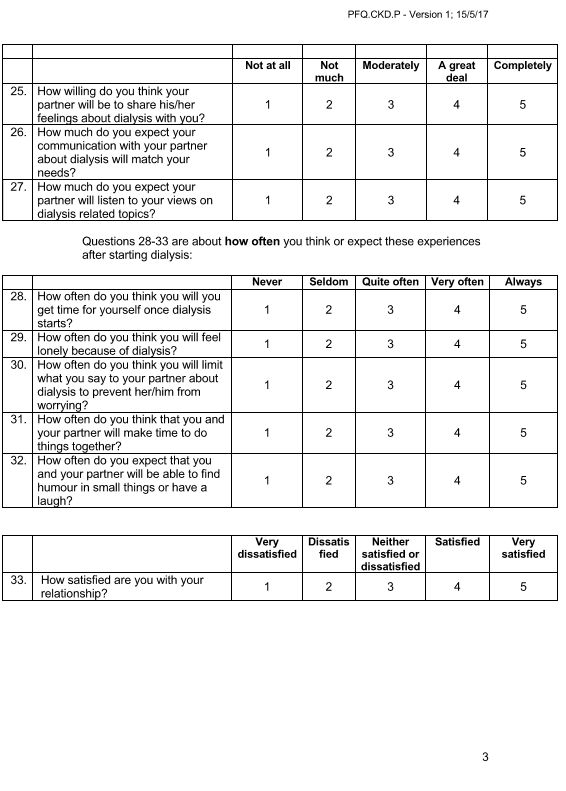


*SDQ – Pre-dialysis – Partner version*


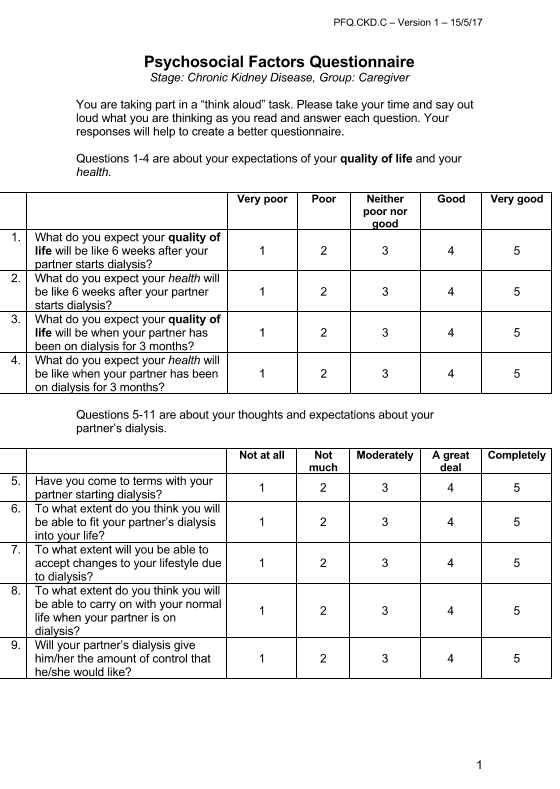


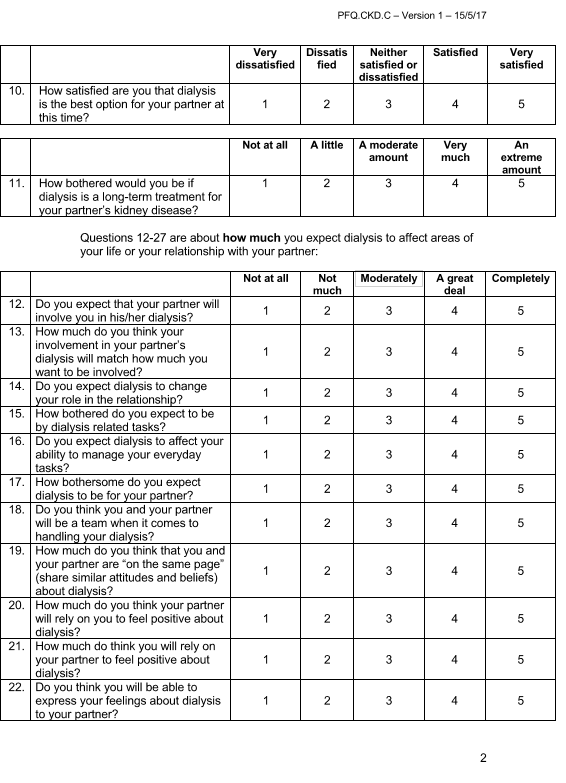


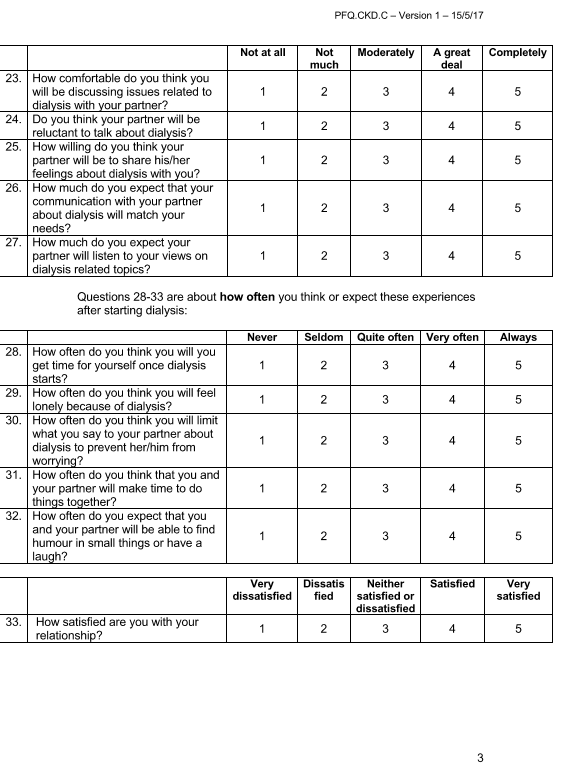


*SDQ – Dialysis – Patient version*


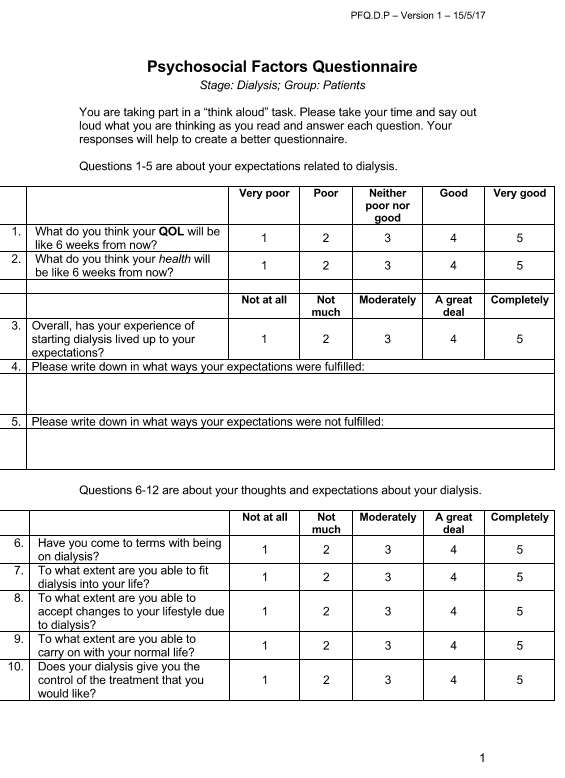


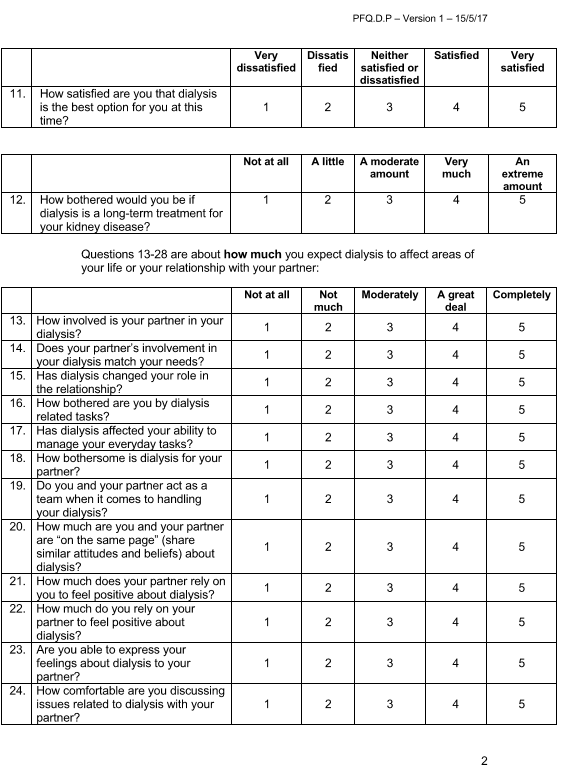


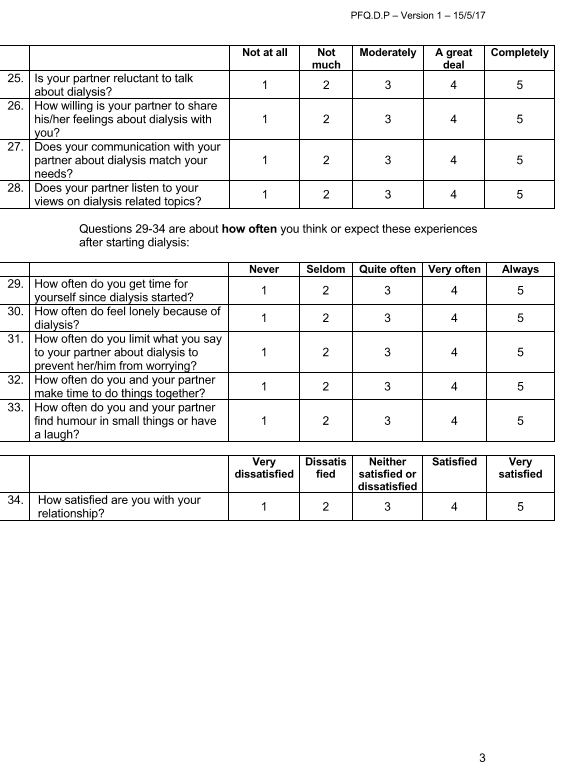


*SDQ – Dialysis – Partner version*


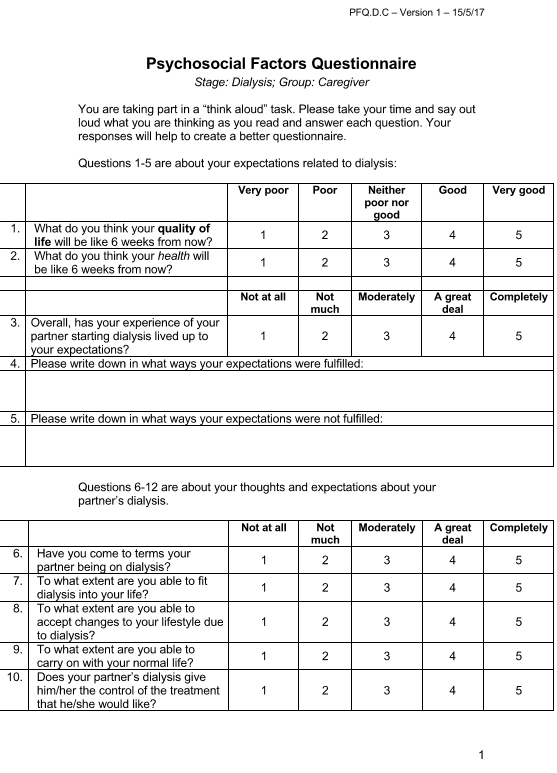


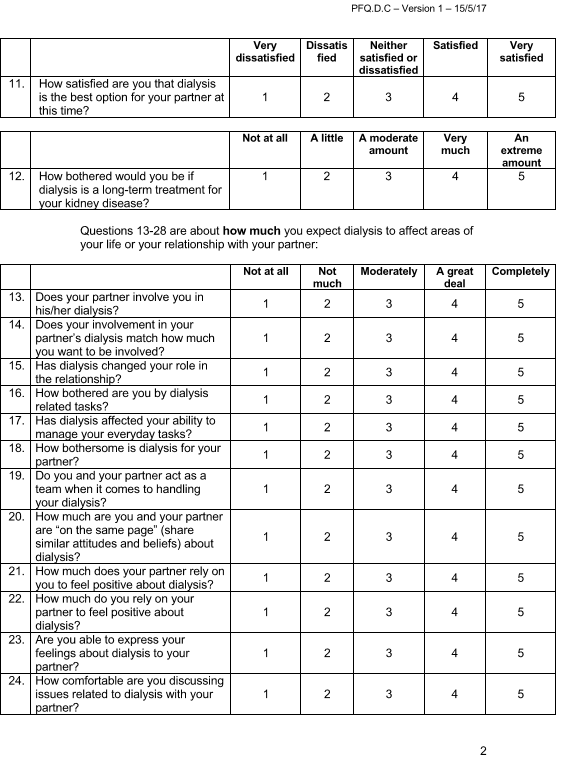


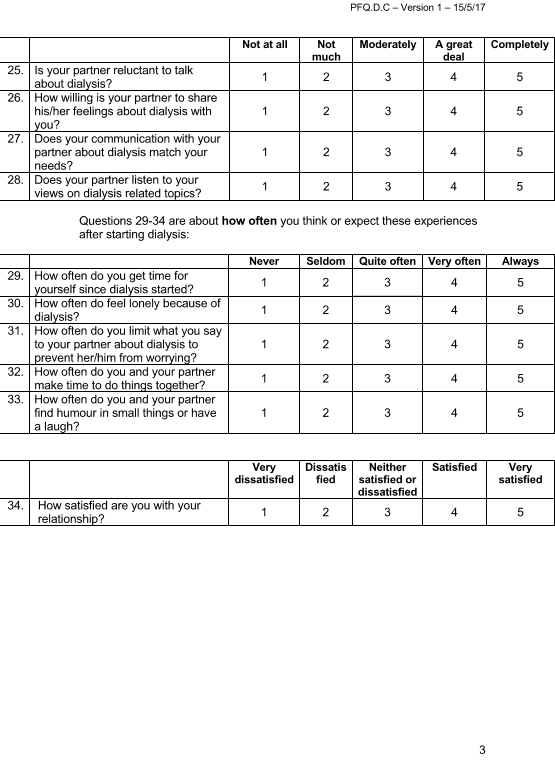

Supplement: Supplementary file 2 — Additional file 2. Developing versions of the Starting Dialysis Questionnaire. [file 12955_2020_1610_MOESM2_ESM.docx]
