# Supplementary material for: Development of a measure for patients preparing to start dialysis and their partners: The Starting Dialysis Questionnaire (SDQ)
Source: Health Qual Life Outcomes. 2020 Nov 7;18:358. doi: 10.1186/s12955-020-01610-x (PMC7648298; doi:10.1186/s12955-020-01610-x)
Supplement: Supplementary file 3 — Additional file 3. Chart showing original questions assessed in cognitive interviews, actions and final questions. [file 12955_2020_1610_MOESM3_ESM.docx]

###### Additional file 3

*Chart showing original items assessed in cognitive interviews, actions and final questions*

| **Item # by Patient Version** | | **Original core question** | **Actions** | **Final questions** |  |
| --- | --- | --- | --- | --- | --- |
| *Pre-D* | *Dialysis* |  |  |  |  |
| 1. | 1. | What do you expect your **QOL** will be like 6 weeks from now? [question phrasing on pre-dialysis version]  What do you think your **QOL** will be like 6 weeks from now? | QOL is a personally defined term and that was deemed acceptable.  On partners’ questionnaire packs, use emphasis on the words ‘your’ to highlight that we are interested in their experiences and thoughts.  In the instructions, state the time frame for all of these questions (i.e., We ask that you think about your life **in the last two weeks**).  Change phrasing from ‘what do you expect’ to ‘what do you think.’  Included. | In 6 weeks, what do you think your **quality of life** will be like? |  |
| 2. | - | [on pre-dialysis version only]  What do you expect your **QOL** will be like 12 weeks from now? | Changed phrasing from ‘12 weeks’ to ‘3 months.‘  Change phrasing from ‘what do you expect’ to ‘what do you think.’ | In 3 months, what do you think your **quality of life** will be like? |  |
| 3. | 2. | What do you expect your *health* will be like 6 weeks from now?  What do you think your *health* will be like 6 weeks from now? | Emotional health reported as important by participants and viewed differently from overall health, especially in early dialysis.  Change phrasing from ‘what do you expect’ to ‘what do you think.’  Included. | In 6 weeks, what do you think your *physical* *health* will be like?  In 3 months, what do you think your *emotional health* will be like? |  |
| 4. | - | [on pre-dialysis version only]  What do you expect your *health* will be like 12 weeks from now? | Changed phrasing from ‘12 weeks’ to ‘3 months’ and ‘what do you expect’ to ‘what do you think.’ | In 3 months, what do you think your *physical* *health* will be like?  In 3 months, what do you think your *emotional health* will be like? |  |
| - | 3. | [No expectation question on pre-dialysis versions]  Overall, has your experience of starting dialysis lived up to your expectations? | At pre-dialysis, add a question about expectations.    At follow-ups (after patient has started dialysis) remove ‘lived up.’  Included. | How would you rate your expectations of dialysis?  How much has dialysis met your expectations? |  |
| - | 4. | [No open-ended expectation question on pre-dialysis versions]  Please write down in what ways your expectations were fulfilled: | Included. | Please write down in what ways you expect dialysis will benefit you or your partner:  Please write down in what ways your expectations have been fulfilled: |  |
| - | 5. | [No open-ended expectation question on pre-dialysis versions]  Please write down in what ways your expectations were not fulfilled: | Included. | Please write down in what ways you expect dialysis will **not** benefit you or your partner:  Please write down in what ways your expectations have **not** been fulfilled: |  |
| 5. | 6. | Have you come to terms with starting dialysis?  Have you come to terms with being on dialysis? | Changed the stem of the question (i.e., the initial wording; e.g., ‘have you’ changed to ‘how much’).  Included. | How much have you come to terms with starting dialysis?  How much have you come to terms with being on dialysis? |  |
| 6. | 7. | To what extent do you think you will be able to fit dialysis into your life?  To what extent are you able to fit dialysis into your life? | Deleted. |  |  |
| 7. | 8. | To what extent will you be able to accept changes to your lifestyle due to dialysis?  To what extent are you able to accept changes to your lifestyle due to dialysis? | Deleted. |  |  |
| 8. | 9. | To what extent do you think you will be able to carry on with your normal life when on dialysis?  To what extent are you able to carry on with your normal life? | Changed ‘normal’ to ‘daily.’  Included. | To what extent do you think you will be able to carry on with your daily life when you start dialysis?  To what extent have you been able to carry on with your daily life since starting dialysis? |  |
| 9. | 10. | Will your dialysis give you the control of the treatment that you would like?  Does your dialysis give you the control of the treatment that you would like? | Deleted ‘of the treatment’  Changed the stem of the question.  Included. | To what extent do you think you will have the control of dialysis that you would like?  To what extent do you have the control of dialysis that you would like? |  |
| 10. | 11. | [same question on both versions]  How satisfied are you that dialysis is the best option for you at this time? | Included. | How satisfied are you that dialysis is the best option for you at this time?  How satisfied are you that dialysis is the best option for you at this time? |  |
| 11. | 12. | [same question on both versions]  How bothered would you be if dialysis is a long-term treatment for your kidney disease? | No other participants had difficulty with the item or disliked the word’ bothered.’  The scoring was flipped to keep it in line with the other items.  Included. | How bothered would you be if dialysis became a long-term treatment for your kidney disease?  How bothered would you be if dialysis became a long-term treatment for your kidney disease? |  |
| 12. | 13. | How much do you expect that your partner will be involved in your dialysis?  How involved is your partner in your dialysis? | Changed the stem of the question.  Included. | How much do you expect that your partner will be involved in your dialysis?  How much is your partner involved in your dialysis? |  |
| 13. | 14. | Do you expect that your partner’s involvement in your dialysis will match your needs?  Does your partner’s involvement in your dialysis match your needs? | Included. | How much do you think your partner’s involvement in your dialysis will match your needs?  How much does your partner’s involvement in your dialysis match your needs? |  |
| 14. | 15. | Do you expect dialysis to change your role in the relationship?  Has dialysis changed your role in the relationship? | Other participants stated ‘role’ was acceptable.  Changed the stem of the question.  Included. | How much do you expect dialysis will change your role in the relationship?  How much has dialysis changed your role in the relationship? |  |
| 15. | 16. | How bothered do you expect to be by dialysis related tasks?  How bothered are you by dialysis related tasks? | Moved to different section of the questionnaire.  Included. | How much do you think you will be bothered by dialysis?  How much are you bothered by dialysis? |  |
| 16. | 17. | Do you expect dialysis to affect your ability to manage your everyday tasks?  Has dialysis affected your ability to manage your everyday tasks? | Deleted. |  |  |
| 17. | 18. | How bothersome do you expect dialysis to be for your partner?  How bothersome is dialysis for your partner? | Moved to different section of the questionnaire.  Included. | How bothersome do you expect dialysis to be for your partner?  How bothersome is dialysis for your partner? |  |
| 18. | 19. | Do you think you and your partner will be a team when it comes to handling your dialysis?  Do you and your partner act as a team when it comes to handling your dialysis? | Changed the stem of the question.  Included. | How much do you think you and your partner will act as a team when it comes to handling your dialysis?  How much do you and your partner act as a team when it comes to handling dialysis? |  |
| 19. | 20. | How much do you think that you and your partner are “on the same page” (share similar attitudes and beliefs) about dialysis?  How much are you and your partner are “on the same page” (share similar attitudes and beliefs) about dialysis? | Included. | How much do you think that you and your partner will be “on the same page” (share similar views) about dialysis?  How much are you and your partner are “on the same page” (share similar views) about dialysis? |  |
| 20. | 21. | How much do you think your partner will rely on you to feel positive about dialysis?  How much does your partner rely on you to feel positive about dialysis? | Change question to directly ask about their own positivity.  Included. | How positive do you think you will be about dialysis?  How positive are you about dialysis? |  |
| 21. | 22. | How much do think you will rely on your partner to feel positive about dialysis?  How much do you rely on your partner to feel positive about dialysis? | Change question to directly ask about their own positivity.  Included. | How positive do you think your partner will be about dialysis?  How positive is your partner towards dialysis? |  |
| 22. | 23. | Do you think you will be able to express your feelings about dialysis to your partner?  Are you able to express your feelings about dialysis to your partner? | Changed the stem of the question and added emphasis to ‘your’ and ‘feelings’ to maintain focus on participant’s own views and distinguish between questions.  Included. | How well do you think you will be able to express your *feelings* about dialysis to your partner?  How well are you able to express your *feelings* about dialysis to your partner? |  |
| 23. | 24. | How comfortable do you think you will be discussing issues related to dialysis with your partner?  How comfortable are you discussing issues related to dialysis with your partner? | To highlight distinction between questions, emphasis added on key words.  Included. | How comfortable do you think you will be discussing *issues* related to dialysis with your partner?  How comfortable are you discussing *issues* related to dialysis with your partner? |  |
| 24. | 25. | Do you think your partner will be reluctant to talk about dialysis?  Is your partner reluctant to talk about dialysis? | Emphasis added and question re-phrased.  Included. | How comfortable do you think your partner will be to talk about dialysis-related *issues*?  How comfortable is your partner talking about dialysis-related *issues*? |  |
| 25. | 26. | How willing do you think your partner will be to share his/her feelings about dialysis with you?  How willing is your partner to share his/her feelings about dialysis with you? | Emphasis added.  Included. | How willing do you think your partner will be to share his/her *feelings* about dialysis with you?  How willing is your partner to share his/her *feelings* about dialysis with you? |  |
| 26. | 27. | How much do you expect your communication with your partner about dialysis will match your needs?  Does your communication with your partner about dialysis match your needs? | Deleted. |  |  |
| 27. | 28. | How much do you expect your partner will listen to your views on dialysis related topics?  Does your partner listen to your views on dialysis related topics? | Changed the stem of the question.  Included. | How much do you think that your partner will listen to your views on dialysis related topics?  How much does your partner listen to your views on dialysis related topics? |  |
| 28. | 29. | How often do you think you will you get time for yourself once dialysis starts?  How often do you get time for yourself since dialysis started? | Question removed from patient versions.  Included. | How often do you think you will you get time for yourself once dialysis starts?  How often do you get time for yourself since dialysis started? |  |
| 29. | 30. | How often do you think you will feel lonely because of dialysis?  How often do you feel lonely because of dialysis? | Added a question on isolation.  Included. | How often do you think you will feel lonely because of dialysis?  How often do you feel lonely because of dialysis?  How often do you think you will feel isolated because of dialysis?  How often do you feel isolated because of dialysis? |  |
| 30. | 31. | How often do you think you will limit what you say to your partner about dialysis to prevent her/him from worrying?  How often do you limit what you say to your partner about dialysis to prevent her/him from worrying? | Deleted. |  |  |
| 31. | 32. | How often do you think that you and your partner will make time to do things together?  How often do you and your partner make time to do things together? | Changed phrasing.  Included. | How often do you think that you and your partner will do activities you enjoy together?  How often do you and your partner do activities you enjoy together? |  |
| 32. | 33. | How often do you expect that you and your partner will be able to find humour in small things or have a laugh?  How often do you and your partner find humour in small things or have a laugh? | Included. | How often do you expect that you and your partner will be able to find humour in small things or have a laugh?  How often do you and your partner find humour in small things or have a laugh? |  |
| 33. | 34. | [same question on both versions]  How satisfied are you with your relationship? | Separated spatially in questionnaire with reminder to think about their life over the last two weeks. | How satisfied are you with your relationship? |  |
| Note. Pre-D=pre-dialysis version of the Starting Dialysis Questionnaire (SDQ). The items presented in the table use the phrasing in the patient versions. The partner versions differ slightly in their wording but address the same topics. The partner items assessed in the cognitive interviews are in Additional File 2 and the final partner version of the SDQ are in Additional File 4.  Response scales with their coordinating question number:  1=Much worse than now, 2=Worse than now, 3=The same, 4=A little better than now, 5=Much better than now – Q1-6  1=Very low, 2=low, 3=No expectations, 4=High, 5=Very high – Q7  1=Not at all, 2=Not much, 3=Moderately, 4=A great deal, 5=Completely – Q10-14, Q17-28  1=Very dissatisfied, 2=Dissatisfied, 3=Neither satisfied or dissatisfied, 4=Satisfied, 5=Very satisfied – Q15, Q34  1=An extreme amount, 2=Very much, 3=A moderate amount, 4=A little, 5=Not at all – Q16  1=Never, 2=Seldom, 3=Quite often, 4=Very often, 5=Always – Q29-33  Response scales taken from “Designing response scales for cross-cultural use in health care: Data from the development of the UK WHOQOL” by S. M. Skevington and C. Tucker, 1999, *British Journal of Medical Psychology, 72*(1), 51-61. | | | | | |
