# Supplementary material for: Development of a measure for patients preparing to start dialysis and their partners: The Starting Dialysis Questionnaire (SDQ)
Source: Health Qual Life Outcomes. 2020 Nov 7;18:358. doi: 10.1186/s12955-020-01610-x (PMC7648298; doi:10.1186/s12955-020-01610-x)
Supplement: Supplementary file 4 — Additional file 4. Final pre-dialysis versions (patient and partner) of the Starting Dialysis Questionnaire. [file 12955_2020_1610_MOESM4_ESM.doc]

**PRE-DIALYSIS**


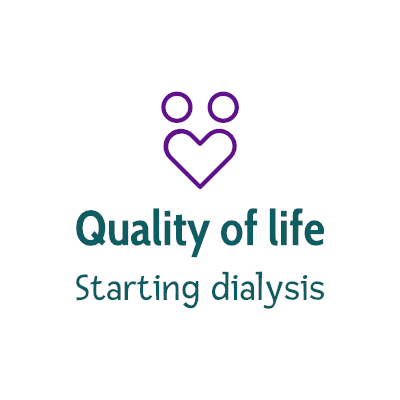


QUESTIONNAIRE

PACK

**PATIENT VERSION**

**Instructions**

This questionnaire asks how you feel about **your** quality of life, health and other areas of your life.

**Please answer all the questions**.

If you are unsure about which response to give to a question, please choose the **ONE** that appears most appropriate. This can often be your first response.

You should **circle** the number that best fits your response.

Please keep in mind your standards, hopes, pleasures and concerns.

We ask that you think about your life **in the last two weeks**.

Please read each question, assess your feelings,

and **circle** the number on the scale for each question that gives the best answer for you.


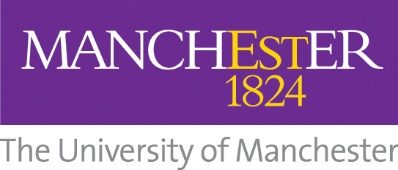


IRAS ID: 226463 Questionnaires Patients T1.V2

06/10/17

**Personal Information**

Please circle your answer.

1. Gender:

1 MALE

2 FEMALE

1. Age: _________ YEARS
2. Date of birth: ___________ (DAY/ MONTH/ YEAR)
3. What is your relationship status?

1 MARRIED

2 CIVIL PARTNERSHIP

3 LIVING TOGETHER

4 DATING

5 ENGAGED

6 OTHER: _______________________________________________

1. What is your highest level of education?
2. NONE
3. PRIMARY SCHOOL
4. SECONDARY SCHOOL
5. COLLEGE OR TRAINING CERTIFICATION
6. UNIVERSITY – UNDERGRADUATE

6 UNIVERSITY – POSTGRADUATE

1. How would you describe your ethnic group:

White

1 BRITISH 2 EUROPEAN 3 OTHER: ___________________________

Black

4 BRITISH 5 AFRICAN 6 CARRIBEAN 7 OTHER: _____________________

Asian

8 INDIAN 9 PAKISTANI 10 CHINESE 11 OTHER: ___________

Other

12 Arab 13 Mixed/Multiple Ethnic Groups: __________________________

1. How would you describe your employment status?
2. RETIRED
3. WORKING FULL-TIME
4. WORKING PART-TIME
5. UNABLE TO WORK
6. DO NOT WORK

**Starting Dialysis Questionnaire**

The following set of questions is about your expectations of your quality of life and your health **in the future***.*

|  |  | **Much worse than now** | **Worse**  **than now** | **The same** | **A little better than now** | **Much better than now** |
| --- | --- | --- | --- | --- | --- | --- |
| 1 | In 6 weeks, what do you think your **quality of life** will be like? | 1 | 2 | 3 | 4 | 5 |
| 2 | In 6 weeks, what do you think your *physical* *health* will be like? | 1 | 2 | 3 | 4 | 5 |
| 3 | In 6 weeks, what do you think your *emotional health* will be like? | 1 | 2 | 3 | 4 | 5 |
| 4 | In 3 months, what do you think your **quality of life** will be like? | 1 | 2 | 3 | 4 | 5 |
| 5 | In 3 months, what do you think your *physical* *health* will be like? | 1 | 2 | 3 | 4 | 5 |
| 6 | In 3 months, what do you think your *emotional health* will be like? | 1 | 2 | 3 | 4 | 5 |

|  |  | **Very low** | **Low** | **No expectations** | **High** | **Very high** |
| --- | --- | --- | --- | --- | --- | --- |
| 7 | How would you rate your expectations of dialysis? | 1 | 2 | 3 | 4 | 5 |

Some people have expectations of dialysis, and some people do not.

To give us a better idea of what patients think about dialysis before starting on it, could you please write down any thoughts you have on the following:

| 8 | Please write down in what ways you expect dialysis will benefit you or your partner: |
| --- | --- |
|  |  |
| 9 | Please write down in what ways you expect it will **not** benefit you or your partner: |
|  |  |

The following questions are about your thoughts and expectations about dialysis.

|  |  | **Not at all** | **Not much** | **Moderately** | **A great deal** | **Completely** |
| --- | --- | --- | --- | --- | --- | --- |
| 10 | How much have you come to terms with starting dialysis? | 1 | 2 | 3 | 4 | 5 |
| 11 | To what extent do you think you will be able to carry on with your daily life when you start dialysis? | 1 | 2 | 3 | 4 | 5 |
| 12 | How much do you think you will be bothered by dialysis? | 1 | 2 | 3 | 4 | 5 |
| 13 | How bothersome do you expect dialysis to be for your partner? | 1 | 2 | 3 | 4 | 5 |
| 14 | To what extent do you think you will have the control of dialysis that you would like? | 1 | 2 | 3 | 4 | 5 |

|  |  | **Very dissatisfied** | **Dissatisfied** | **Neither satisfied or dissatisfied** | **Satisfied** | **Very satisfied** |
| --- | --- | --- | --- | --- | --- | --- |
| 15 | How satisfied are you that dialysis is the best option for you at this time? | 1 | 2 | 3 | 4 | 5 |

|  |  | **An extreme amount** | **Very much** | **A moderate amount** | **A little** | **Not at all** |
| --- | --- | --- | --- | --- | --- | --- |
| 16 | How bothered would you be if dialysis became a long-term treatment for your kidney disease? | 1 | 2 | 3 | 4 | 5 |

The following questions are about **how much** you expect dialysis to affect areas

of your life or your relationship with your partner.

|  |  | **Not at all** | **Not much** | **Moderately** | **A great deal** | **Completely** |
| --- | --- | --- | --- | --- | --- | --- |
| 17 | How much do you expect that your partner will be involved in your dialysis? | 1 | 2 | 3 | 4 | 5 |
| 18 | How much do you think your partner’s involvement in your dialysis will match your needs? | 1 | 2 | 3 | 4 | 5 |
| 19 | How much do you expect dialysis will change your role in the relationship? | 1 | 2 | 3 | 4 | 5 |
| 20 | How much do you think you and your partner will act as a team when it comes to handling your dialysis? | 1 | 2 | 3 | 4 | 5 |
| 21 | How much do you think that you and your partner will be “on the same page” (share similar views) about dialysis? | 1 | 2 | 3 | 4 | 5 |
| 22 | How positive do you think you will be about dialysis? | 1 | 2 | 3 | 4 | 5 |
| 23 | How positive do you think your partner will be about dialysis? | 1 | 2 | 3 | 4 | 5 |
| 24 | How well do you think you will be able to express your *feelings* about dialysis to your partner? | 1 | 2 | 3 | 4 | 5 |
| 25 | How comfortable do you think you will be discussing *issues* related to dialysis with your partner? | 1 | 2 | 3 | 4 | 5 |
| 26 | How comfortable do you think your partner will be to talk about dialysis-related *issues*? | 1 | 2 | 3 | 4 | 5 |
| 27 | How willing do you think your partner will be to share his/her *feelings* about dialysis with you? | 1 | 2 | 3 | 4 | 5 |
| 28 | How much do you think that your partner will listen to your views on dialysis related topics? | 1 | 2 | 3 | 4 | 5 |

The following questions about **how often** you think or expect these experiences after starting dialysis.

|  |  | **Never** | **Seldom** | **Quite often** | **Very often** | **Always** |
| --- | --- | --- | --- | --- | --- | --- |
| 30 | How often do you think you will feel lonely because of dialysis? | 1 | 2 | 3 | 4 | 5 |
| 31 | How often do you think you will feel isolated because of dialysis? | 1 | 2 | 3 | 4 | 5 |
| 32 | How often do you think that you and your partner will do activities you enjoy together? | 1 | 2 | 3 | 4 | 5 |
| 33 | How often do you expect that you and your partner will be able to find humour in small things or have a laugh? | 1 | 2 | 3 | 4 | 5 |

The following question asks you to think about **how satisfied** you are with your life **over the**

**last two weeks.**

|  |  | **Very dissatisfied** | **Dissatisfied** | **Neither satisfied or dissatisfied** | **Satisfied** | **Very satisfied** |
| --- | --- | --- | --- | --- | --- | --- |
| 34 | How satisfied are you with your relationship? | 1 | 2 | 3 | 4 | 5 |

**Thank you for answering these questions. Your contribution is greatly appreciated.**

Is there anything else you would like to tell us about?

Also, any comments that you think may help us to understand dialysis patients

and their partners would be most appreciated.

Please feel free to use this space for this purpose.

**To be completed by the person who gives you the questionnaire pack:**

Participant Id: _____________________________ Renal Centre: _____________________________

Date completed: ______________ (dd/mm/yy) Approximate time to complete: __________ (minutes)

How was the questionnaire completed:

_____ Completed without assistance from researcher or investigator

_____ Assisted by researcher or investigator (read aloud)

_____ Other – please provide details:____________________________

Where was the questionnaire completed:

____ At a clinical appointment

____ Before a dialysis session

____ During a dialysis session

____ At home

____ Other: ______________________________________

**PRE-DIALYSIS**


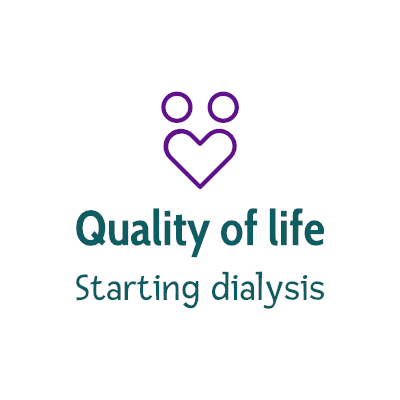


QUESTIONNAIRE

PACK

**PARTNER VERSION**

**Instructions**

This questionnaire asks how you feel about **your** quality of life, health and other areas of your life.

Your needs matter even as your partner prepares for dialysis.

By completing this questionnaire, you will help us understand your experience

and how we can better support you both.

**Please answer all the questions**.

If you are unsure about which response to give to a question, please choose the **ONE** that appears most appropriate. This can often be your first response.

You should **circle** the number that best fits your response.

Please keep in mind your standards, hopes, pleasures and concerns.

We ask that you think about your life **in the last two weeks**.

Please read each question, assess your feelings,

and **circle** the number on the scale for each question that gives the best answer for you.


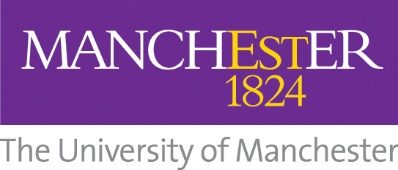


IRAS ID: 226463 Questionnaires Partners T1.V2

Date: 06/10/17

**Personal Information**

Please circle your answer.

1. Gender:

1 MALE

2 FEMALE

1. Age: _________ YEARS
2. Date of birth: ___________ (DAY/ MONTH/ YEAR)
3. What is your relationship status?

1 MARRIED

2 CIVIL PARTNERSHIP

3 LIVING TOGETHER

4 DATING

5 ENGAGED

6 OTHER: _______________________________________________

1. What is your highest level of education?
2. NONE
3. PRIMARY SCHOOL
4. SECONDARY SCHOOL
5. COLLEGE OR TRAINING CERTIFICATION
6. UNIVERSITY – UNDERGRADUATE

6 UNIVERSITY – POSTGRADUATE

1. How would you describe your ethnic group:

White

1 BRITISH 2 EUROPEAN 3 OTHER: ___________________________

Black

4 BRITISH 5 AFRICAN 6 CARRIBEAN 7 OTHER: _____________________

Asian

8 INDIAN 9 PAKISTANI 10 CHINESE 11 OTHER: ___________

Other

12 Arab 13 Mixed/Multiple Ethnic Groups: __________________________

1. How would you describe your employment status?
2. RETIRED
3. WORKING FULL-TIME
4. WORKING PART-TIME
5. UNABLE TO WORK
6. DO NOT WORK

**Starting Dialysis Questionnaire**

The following set of questions is about your expectations of your quality of life and your health **in the future***.*

|  |  | **Much worse than now** | **Worse**  **than now** | **The same** | **A little better than now** | **Much better than now** |
| --- | --- | --- | --- | --- | --- | --- |
| 1 | In 6 weeks, what do you think your **quality of life** will be like? | 1 | 2 | 3 | 4 | 5 |
| 2 | In 6 weeks, what do you think your *physical* *health* will be like? | 1 | 2 | 3 | 4 | 5 |
| 3 | In 6 weeks, what do you think your *emotional health* will be like? | 1 | 2 | 3 | 4 | 5 |
| 4 | In 3 months, what do you think your **quality of life** will be like? | 1 | 2 | 3 | 4 | 5 |
| 5 | In 3 months, what do you think your *physical* *health* will be like? | 1 | 2 | 3 | 4 | 5 |
| 6 | In 3 months, what do you think your *emotional health* will be like? | 1 | 2 | 3 | 4 | 5 |

|  |  | **Very low** | **Low** | **No expectations** | **High** | **Very high** |
| --- | --- | --- | --- | --- | --- | --- |
| 7 | How would you rate your expectations of dialysis? | 1 | 2 | 3 | 4 | 5 |

Some people have expectations of dialysis, and some people do not.

To give us a better idea of what partners think about dialysis before their patient-partner starts on it, could you please write down any thoughts you have on the following:

| 8 | Please write down in what ways you expect dialysis will benefit you or your partner: |
| --- | --- |
|  |  |
| 9 | Please write down in what ways you expect it will **not** benefit you or your partner: |
|  |  |

The following questions are about your thoughts and expectations about your partner’s dialysis.

|  |  | **Not at all** | **Not much** | **Moderately** | **A great deal** | **Completely** |
| --- | --- | --- | --- | --- | --- | --- |
| 10 | How much have you come to terms with your partner starting dialysis? | 1 | 2 | 3 | 4 | 5 |
| 11 | To what extent do you think you will be able to carry on with your daily life when your partner is on dialysis? | 1 | 2 | 3 | 4 | 5 |
| 12 | How much do you think you will be bothered by dialysis? | 1 | 2 | 3 | 4 | 5 |
| 13 | How bothersome do you expect dialysis to be for your partner? | 1 | 2 | 3 | 4 | 5 |
| 14 | To what extent do you think your partner will have the control of dialysis that he/she would like? | 1 | 2 | 3 | 4 | 5 |

|  |  | **Very dissatisfied** | **Dissatisfied** | **Neither satisfied or dissatisfied** | **Satisfied** | **Very satisfied** |
| --- | --- | --- | --- | --- | --- | --- |
| 15 | How satisfied are you that dialysis is the best option for your partner at this time? | 1 | 2 | 3 | 4 | 5 |

|  |  | **An extreme amount** | **Very much** | **A moderate amount** | **A little** | **Not at all** |
| --- | --- | --- | --- | --- | --- | --- |
| 16 | How bothered would you be if dialysis became a long-term treatment for your partner’s kidney disease? | 1 | 2 | 3 | 4 | 5 |

The following questions are about **how much** you expect dialysis to affect areas of your life or your relationship with your partner. We ask that you think about your life **in the last two weeks**.

|  |  | **Not at all** | **Not much** | **Moderately** | **A great deal** | **Completely** |
| --- | --- | --- | --- | --- | --- | --- |
| 17 | How much do you expect that your partner will involve you in his/her dialysis? | 1 | 2 | 3 | 4 | 5 |
| 18 | How much do you think your involvement in your partner’s dialysis will match how much you want to be involved? | 1 | 2 | 3 | 4 | 5 |
| 19 | How much do you expect dialysis will change your role in the relationship? | 1 | 2 | 3 | 4 | 5 |
| 20 | How much do you think you and your partner will act as a team when it comes to handling dialysis? | 1 | 2 | 3 | 4 | 5 |
| 21 | How much do you think that you and your partner will be “on the same page” (share similar views) about dialysis? | 1 | 2 | 3 | 4 | 5 |
| 22 | How positive do you think you will be about dialysis? | 1 | 2 | 3 | 4 | 5 |
| 23 | How positive do you think your partner will be about dialysis? | 1 | 2 | 3 | 4 | 5 |
| 24 | How well do you think you will be able to express your *feelings* about dialysis to your partner? | 1 | 2 | 3 | 4 | 5 |
| 25 | How comfortable do you think you will be discussing *issues* related to dialysis with your partner? | 1 | 2 | 3 | 4 | 5 |
| 26 | How comfortable do you think your partner will be to talk about dialysis-related *issues*? | 1 | 2 | 3 | 4 | 5 |
| 27 | How willing do you think your partner will be to share his/her *feelings* about dialysis with you? | 1 | 2 | 3 | 4 | 5 |
| 28 | How much do you think that your partner will listen to your views on dialysis related topics? | 1 | 2 | 3 | 4 | 5 |

The following questions about **how often** you think or expect these experiences after starting dialysis.

We ask that you think about your life **in the last two weeks**.

|  |  | **Never** | **Seldom** | **Quite often** | **Very often** | **Always** |
| --- | --- | --- | --- | --- | --- | --- |
| 29 | How often do you think you will you get time for yourself once dialysis starts? |  |  |  |  |  |
| 30 | How often do you think you will feel lonely because of dialysis? | 1 | 2 | 3 | 4 | 5 |
| 31 | How often do you think you will feel isolated because of dialysis? | 1 | 2 | 3 | 4 | 5 |
| 32 | How often do you think that you and your partner will do activities you enjoy together? | 1 | 2 | 3 | 4 | 5 |
| 33 | How often do you expect that you and your partner will be able to find humour in small things or have a laugh? | 1 | 2 | 3 | 4 | 5 |

The following question asks you to think about **how satisfied** you are with your life **over the**

**last two weeks.**

|  |  | **Very dissatisfied** | **Dissatisfied** | **Neither satisfied or dissatisfied** | **Satisfied** | **Very satisfied** |
| --- | --- | --- | --- | --- | --- | --- |
| 34 | How satisfied are you with your relationship? | 1 | 2 | 3 | 4 | 5 |

**Thank you for answering these questions. Your contribution is greatly appreciated.**

Is there anything else you would like to tell us about?

Also, any comments that you think may help us to understand dialysis patients

and their partners would be most appreciated.

Please feel free to use this space for this purpose.

**To be completed by the person who gives you the questionnaire pack:**

Participant Id: _____________________________ Renal Centre: _____________________________

Date completed: ______________ (dd/mm/yy) Approximate time to complete: __________ (minutes)

How was the questionnaire completed:

_____ Completed without assistance from researcher or investigator

_____ Assisted by researcher or investigator (read aloud)

_____ Other – please provide details:____________________________

Where was the questionnaire completed:

____ At a clinical appointment

____ Before a dialysis session

____ During a dialysis session

____ At home

____ Other: ______________________________________

**DIALYSIS**

QUESTIONNAIRE

PACK


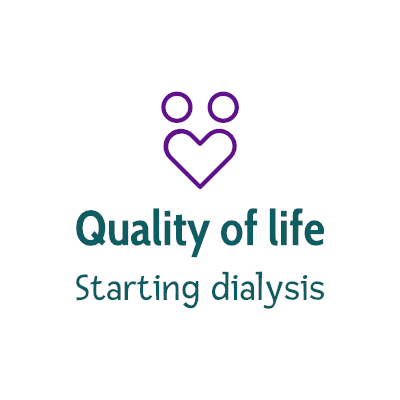


**PATIENT VERSION**

**Instructions**

Thank you for your participation in this study.

This questionnaire asks how you feel about your quality of life,

health and other areas of your life.

**Please answer all the questions**.

If you are unsure about which response to give to a question, please choose the **ONE** that appears most appropriate. This can often be your first response.

You should **circle** the number that best fits your response.

Please keep in mind your standards, hopes, pleasures and concerns.

We ask that you think about your life **in the last two weeks**.

Please read each question, assess your feelings,

and **circle** the number on the scale for each question that gives the best answer for you.


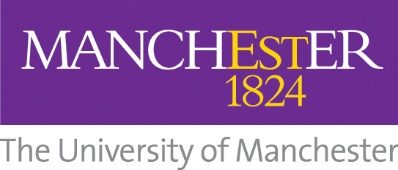


IRAS ID: 226463 Questionnaires Patients T2.V2

06/10/17

**Dialysis Information**

Please circle your answer.

Since you started dialysis, have you experienced any of the following?

1. Change of location where you have dialysis?
   1. Yes
   2. No

If you circled yes, where do you receive your dialysis treatment now?

________________________________________________­­­­­­­­­­­­­­­­­­­­­­­­­­­_____________

1. Change in your access for dialysis (fistula or catheter)?
   1. Yes
   2. No

If you circled yes, please say what changed: _________________________

1. Change in the type of dialysis treatment you receive?
   1. Yes
   2. No

If you circled yes, please say what type you were on before and what you are on now:

________________________________________________________________

1. Have you had to stay in the hospital?
   1. Yes
   2. No

If yes, please say how long you stayed and the nature of the hospitalization:

________________________________________________________________

**Starting Dialysis Questionnaire**

The following set of questions is about your expectations of your quality of life and your health

**in the future***.*

|  |  | **Much worse than now** | **Worse**  **than now** | **The same** | **A little better than now** | **Much better than now** |
| --- | --- | --- | --- | --- | --- | --- |
| 1. | In 6 weeks, what do you think your **quality of life** will be like? | 1 | 2 | 3 | 4 | 5 |
| 2. | In 6 weeks, what do you think your *physical* *health* will be like? | 1 | 2 | 3 | 4 | 5 |
| 3. | In 6 weeks, what do you think your *emotional health* will be like? | 1 | 2 | 3 | 4 | 5 |

|  |  | **Not at all** | **Not much** | **Moderately** | **A great deal** | **Completely** |
| --- | --- | --- | --- | --- | --- | --- |
| 4. | How much has dialysis met your expectations? | 1 | 2 | 3 | 4 | 5 |

To give us a better idea of what patients think about dialysis after they have started on it, could you please write down any thoughts you have on the following:

| 5. | Please write down in what ways your expectations of dialysis have been fulfilled: |
| --- | --- |
|  |  |
| 6. | Please write down in what ways your expectations have **not** been fulfilled: |
|  |  |

The following questions are about your thoughts and attitudes about dialysis.

We ask that you think about these questions in the context of **the last two weeks**

|  |  | **Not at all** | **Not much** | **Moderately** | **A great deal** | **Completely** |
| --- | --- | --- | --- | --- | --- | --- |
| 7. | How much have you come to terms with being on dialysis? | 1 | 2 | 3 | 4 | 5 |
| 8. | To what extent have you been able to carry on with your daily life since starting dialysis? | 1 | 2 | 3 | 4 | 5 |
| 9. | How much are you bothered by dialysis? | 1 | 2 | 3 | 4 | 5 |
| 10. | How bothersome is dialysis for your partner? | 1 | 2 | 3 | 4 | 5 |
| 11. | To what extent do you have the control of dialysis that you would like? | 1 | 2 | 3 | 4 | 5 |

|  |  | **Very dissatisfied** | **Dissatisfied** | **Neither satisfied or dissatisfied** | **Satisfied** | **Very satisfied** |
| --- | --- | --- | --- | --- | --- | --- |
| 12. | How satisfied are you that dialysis is the best option for you at this time? | 1 | 2 | 3 | 4 | 5 |

|  |  | **An extreme amount** | **Very much** | **A moderate amount** | **A little** | **Not at all** |
| --- | --- | --- | --- | --- | --- | --- |
| 13. | How bothered would you be if dialysis became a long-term treatment for your kidney disease? | 1 | 2 | 3 | 4 | 5 |

The following questions are about **how much** dialysis affects areas of your life or your relationship with your partner. We ask that you think about your life **in the last two weeks**.

|  |  | **Not at all** | **Not much** | **Moderately** | **A great deal** | **Completely** |
| --- | --- | --- | --- | --- | --- | --- |
| 14. | How much is your partner involved in your dialysis? | 1 | 2 | 3 | 4 | 5 |
| 15. | How much does your partner’s involvement in your dialysis match your needs? | 1 | 2 | 3 | 4 | 5 |
| 16. | How much has dialysis changed your role in the relationship? | 1 | 2 | 3 | 4 | 5 |
| 17. | How much do you and your partner act as a team when it comes to handling dialysis? | 1 | 2 | 3 | 4 | 5 |
| 18. | How much are you and your partner are “on the same page” (share similar views) about dialysis? | 1 | 2 | 3 | 4 | 5 |
| 19. | How positive are you about dialysis? | 1 | 2 | 3 | 4 | 5 |
| 20. | How positive is your partner towards dialysis? | 1 | 2 | 3 | 4 | 5 |
|  |  |  |  |  |  |  |
|  |  |  |  |  |  |  |
|  |  | **Not at all** | **Not much** | **Moderately** | **A great deal** | **Completely** |
| 21. | How well are you able to express your *feelings* about dialysis to your partner? | 1 | 2 | 3 | 4 | 5 |
| 22. | How comfortable are you discussing *issues* related to dialysis with your partner? | 1 | 2 | 3 | 4 | 5 |
| 23. | How comfortable is your partner talking about dialysis-related *issues*? | 1 | 2 | 3 | 4 | 5 |
| 24. | How willing is your partner to share his/her *feelings* about dialysis with you? | 1 | 2 | 3 | 4 | 5 |
| 25. | How much does your partner listen to your views on dialysis related topics? | 1 | 2 | 3 | 4 | 5 |

The following questions ask about **how often** you experience these feelings or activities since starting dialysis. We ask that you think about your life **in the last two weeks**.

|  |  | **Never** | **Seldom** | **Quite often** | **Very often** | **Always** |
| --- | --- | --- | --- | --- | --- | --- |
| 26. | How often do you feel lonely because of dialysis? | 1 | 2 | 3 | 4 | 5 |
| 27. | How often do you feel isolated because of dialysis? | 1 | 2 | 3 | 4 | 5 |
| 28. | How often do you and your partner do activities you enjoy together? | 1 | 2 | 3 | 4 | 5 |
| 29. | How often do you and your partner find humour in small things or have a laugh? | 1 | 2 | 3 | 4 | 5 |

The following question asks you to think about **how satisfied** you are with aspects of your life **over the last two weeks**.

|  |  | **Very dissatisfied** | **Dissatisfied** | **Neither satisfied or dissatisfied** | **Satisfied** | **Very satisfied** |
| --- | --- | --- | --- | --- | --- | --- |
| 30. | How satisfied are you with your relationship? | 1 | 2 | 3 | 4 | 5 |

**DIALYSIS**


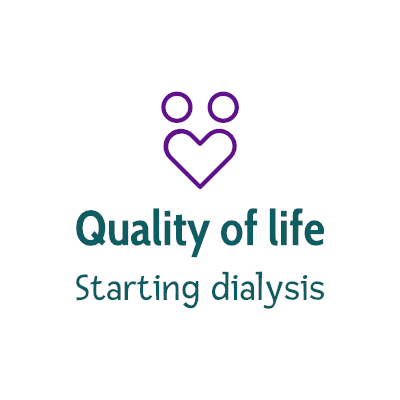


QUESTIONNAIRE

PACK

**PARTNER VERSION**

**Instructions**

Thank you for your continued participation in this study.

This questionnaire asks how you feel about **your** quality of life, health and other areas of your life.

Your needs matter even after your partner has started on dialysis.

By completing this questionnaire, you will help us understand your experience

and how we can better support you both.

**Please answer all the questions**.

If you are unsure about which response to give to a question, please choose the **ONE** that appears most appropriate. This can often be your first response.

You should **circle** the number that best fits your response.

Please keep in mind your standards, hopes, pleasures and concerns.

We ask that you think about your life **in the last two weeks**.

Please read each question, assess your feelings,

and **circle** the number on the scale for each question that gives the best answer for you.


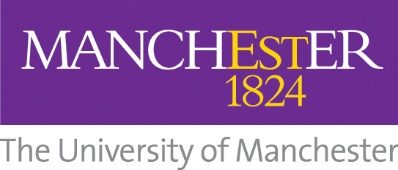


IRAS ID: 226463 Questionnaires Partners T2.V2

Date: 06/10/17

**Starting Dialysis Questionnaire**

The following set of questions is about your expectations of your quality of life and your health

**in the future***.*

|  |  | **Much worse than now** | **Worse**  **than now** | **The same** | **A little better than now** | **Much better than now** |
| --- | --- | --- | --- | --- | --- | --- |
| 1. | In 6 weeks, what do you think your **quality of life** will be like? | 1 | 2 | 3 | 4 | 5 |
| 2. | In 6 weeks, what do you think your *physical* *health* will be like? | 1 | 2 | 3 | 4 | 5 |
| 3. | In 6 weeks, what do you think your *emotional health* will be like? | 1 | 2 | 3 | 4 | 5 |

|  |  | **Not at all** | **Not much** | **Moderately** | **A great deal** | **Completely** |
| --- | --- | --- | --- | --- | --- | --- |
| 4. | How much has dialysis met your expectations? | 1 | 2 | 3 | 4 | 5 |

To give us a better idea of what partners think about dialysis after their patient-partners have started on it, could you please write down any thoughts you have on the following:

| 5. | Please write down in what ways your expectations of dialysis have been fulfilled: |
| --- | --- |
|  |  |
| 6. | Please write down in what ways your expectations have **not** been fulfilled: |
|  |  |

The following questions are about your thoughts and attitudes about your partner’s dialysis.

We ask that you think about these questions in the context of **the last two weeks**.

|  |  | **Not at all** | **Not much** | **Moderately** | **A great deal** | **Completely** |
| --- | --- | --- | --- | --- | --- | --- |
| 7. | How much have you come to terms with your partner being on dialysis? | 1 | 2 | 3 | 4 | 5 |
| 8. | To what extent have you been able to carry on with your daily life since your partner started dialysis? | 1 | 2 | 3 | 4 | 5 |
| 9. | How much are you bothered by dialysis? | 1 | 2 | 3 | 4 | 5 |
| 10. | How bothersome is dialysis for your partner? | 1 | 2 | 3 | 4 | 5 |
| 11. | To what extent does your partner have the control of dialysis that he/she would like? | 1 | 2 | 3 | 4 | 5 |

|  |  | **Very dissatisfied** | **Dissatisfied** | **Neither satisfied or dissatisfied** | **Satisfied** | **Very satisfied** |
| --- | --- | --- | --- | --- | --- | --- |
| 12. | How satisfied are you that dialysis is the best option for your partner at this time? | 1 | 2 | 3 | 4 | 5 |

|  |  | **An extreme amount** | **Very much** | **A moderate amount** | **A little** | **Not at all** |
| --- | --- | --- | --- | --- | --- | --- |
| 13. | How bothered would you be if dialysis became a long-term treatment for your partner’s kidney disease? | 1 | 2 | 3 | 4 | 5 |

The following questions are about **how much** dialysis affects areas of your life or your relationship with your partner. We ask that you think about your life **in the last two weeks**.

|  |  | **Not at all** | **Not much** | **Moderately** | **A great deal** | **Completely** |
| --- | --- | --- | --- | --- | --- | --- |
| 14. | How much does your partner involve you in his/her dialysis? | 1 | 2 | 3 | 4 | 5 |
| 15. | How much does your involvement in your partner’s dialysis match how much you want to be involved? | 1 | 2 | 3 | 4 | 5 |
| 16. | How much has dialysis changed your role in the relationship? | 1 | 2 | 3 | 4 | 5 |
| 17. | How much do you and your partner act as a team when it comes to handling dialysis? | 1 | 2 | 3 | 4 | 5 |
| 18. | How much are you and your partner are “on the same page” (share similar views) about dialysis? | 1 | 2 | 3 | 4 | 5 |
| 19. | How positive are you about dialysis? | 1 | 2 | 3 | 4 | 5 |
|  |  |  |  |  |  |  |
|  |  | **Not at all** | **Not much** | **Moderately** | **A great deal** | **Completely** |
| 20. | How positive is your partner towards dialysis? | 1 | 2 | 3 | 4 | 5 |
| 21. | How well are you able to express your *feelings* about dialysis to your partner? | 1 | 2 | 3 | 4 | 5 |
| 22. | How comfortable are you discussing *issues* related to dialysis with your partner? | 1 | 2 | 3 | 4 | 5 |
| 23. | How comfortable is your partner talking about dialysis-related *issues*? | 1 | 2 | 3 | 4 | 5 |
| 24. | How willing is your partner to share his/her *feelings* about dialysis with you? | 1 | 2 | 3 | 4 | 5 |
| 25. | How much does your partner listen to your views on dialysis related topics? | 1 | 2 | 3 | 4 | 5 |

The following questions ask about **how often** you experience these feelings or activities since starting dialysis. We ask that you think about your life **in the last two weeks**.

|  |  | **Never** | **Seldom** | **Quite often** | **Very often** | **Always** |
| --- | --- | --- | --- | --- | --- | --- |
| 26. | How often do you get time for yourself since dialysis started? | 1 | 2 | 3 | 4 | 5 |
| 27. | How often do you feel lonely because of dialysis? | 1 | 2 | 3 | 4 | 5 |
| 28. | How often do you feel isolated because of dialysis? | 1 | 2 | 3 | 4 | 5 |
| 29. | How often do you and your partner do activities you enjoy together? | 1 | 2 | 3 | 4 | 5 |
| 30. | How often do you and your partner find humour in small things or have a laugh? | 1 | 2 | 3 | 4 | 5 |

The following question asks you to think about **how satisfied** you are with aspects of your life **over the last two weeks**.

|  |  | **Very dissatisfied** | **Dissatisfied** | **Neither satisfied or dissatisfied** | **Satisfied** | **Very satisfied** |
| --- | --- | --- | --- | --- | --- | --- |
| 31. | How satisfied are you with your relationship? | 1 | 2 | 3 | 4 | 5 |
